# LRRC8A critically regulates myofibroblast phenotypes and fibrotic remodeling following myocardial infarction

**DOI:** 10.7150/thno.75200

**Published:** 2022-07-27

**Authors:** Xiyao Chen, Fuyang Zhang, Guangyu Hu, Xing Li, Lin Wang, Congye Li, Cong Huo, Rong Xu, Liming Hou, Ning Wang, Xiaoming Wang

**Affiliations:** 1 Department of Geriatrics, Xijing Hospital, Fourth Military Medical University, Xi'an, 710032 China.; 2 Department of Cardiology, Xijing Hospital, Fourth Military Medical University, Xi'an, 710032 China.

**Keywords:** fibrosis, heart failure, leucine-rich repeat-containing protein 8A, myocardial infarction, myofibroblast.

## Abstract

**Rationale:** The transformation of fibroblasts into activated myofibroblasts is a critical step that results in cardiac fibrosis upon myocardial infarction (MI). Leucine-rich repeat-containing protein-8A (LRRC8A) is a multi-functional protein involved in cell survival, growth, and proliferation, whereas its role in regulating myofibroblast phenotypes and myocardial fibrosis remains unknown.

**Methods:** Conditional myofibroblast-specific *Lrrc8a* knockout mouse models were established by crossing the *Lrrc8a*^flox/flox^ mice with the tamoxifen-inducible periostin-Cre transgenic mice. The involvement of LRRC8A in regulating cardiac fibrosis post-MI and myofibroblast phenotypes induced by transforming growth factor-β1 (TGF-β1) was comprehensively evaluated. The mechanisms underlying LRRC8A regulation of myofibroblast phenotypes were determined by RNA sequencing-driven analysis followed by cause-effect experiments.

**Results:** LRRC8A expression was significantly elevated in the fibrotic tissues and the fibroblasts isolated from the post-MI hearts. Compared with the wild-type (WT) littermates, the specific knockout of LRRC8A in myofibroblasts greatly attenuated myofibroblast transformation, fibrotic remodeling, and ventricular dysfunction after MI. Silencing of LRRC8A expression suppressed, whereas overexpression of LRRC8A enhanced, the pro-fibrotic myofibroblast phenotypes in isolated cardiac fibroblasts upon stimulation with TGF-β1. LRRC8A participated in TGF-β1-induced myofibroblast transformation via activating JAK2-STAT3 signaling. Furthermore, LRRC8A activated the JAK2-STAT3 pathway via its C-terminal leucine-rich repeat-domain (LRRD), directly interacting with growth factor receptor-bound protein 2 (GRB2), an adaptor protein associated with and necessary for tyrosine-phosphorylated JAK2.

**Conclusions:** LRRC8A regulates myofibroblast transformation and cardiac fibrosis following MI. LRRC8A inhibition might be a promising strategy for cardiac fibrosis and heart failure.

## Introduction

Heart failure (HF) is a mortal disease and remains the predominate cause of mortality in the world, presenting a heavy economic and health burden 1. Regardless of etiology, HF generally involves myocardial fibrosis characterized by the activation of quiescent cardiac fibroblasts into myofibroblasts and excessive deposition of extracellular matrix (ECM). Myocardial fibrosis suppresses heart tissue compliance, promotes ventricular dysfunction, and accelerates HF development 2. Despite the importance of cardiac fibrosis in the pathogenesis of HF, the underlying mechanism is largely elusive. There are no effective therapies that target myofibroblasts or their detrimental contributions to HF progression.

Cardiac fibroblasts are involved in both reparative and pathological fibrotic responses following myocardial infarction (MI) 3. In healthy hearts, cardiac fibroblasts are quiescent and their capability for ECM production is limited. In post-ischemic hearts, the loss of cardiomyocytes and the infiltration of inflammatory cells stimulate the secretion of pro-fibrotic cytokines such as transforming growth factor-β1 (TGF-β1) to induce the transformation of the quiescent fibroblasts into the activated myofibroblasts 4. Myofibroblasts are characterized by contractile phenotypes and enrichment in α-smooth muscle actin (α-SMA) 5. Activated myofibroblasts have been identified as the main source of ECM in post-ischemic hearts, and persistent myofibroblast activity results in excessive fibrotic scarring, loss of tissue compliance, and ultimately, the development of HF.

In mammalian cells, leucine-rich repeat-containing protein-8A (LRRC8A), also named as SWELL1, is newly recognized as a membrane protein constituting the volume regulatory anion channel (VRAC) 6-7. A large amount of evidence has demonstrated that LRRC8A is involved in the regulation of multiple cellular processes, including cell survival, growth, proliferation, energy sensing, and immune responses 8-12. These biological effects of LRRC8A seem to be mediated mainly through its anion channel-independent function 8-12. Importantly, deregulated LRRC8A expression or function is tightly associated with a series of diseases, such as obesity, type 2 diabetes mellitus, vascular injury, and malignant cancers 13-16. We recently demonstrated that inhibiting LRRC8A expression in cardiomyocytes reduces angiotensin II-induced ventricular hypertrophy and dysfunction, giving the first evidence that LRRC8A functions as a key regulator of cardiovascular pathology 17. However, the role of LRRC8A in fibroblast-to-myofibroblast activation and cardiac fibrotic remodeling is yet unknown. Considering the significance of LRRC8A in controlling cell fate and phenotypes, we proposed that LRRC8A may participate in myofibroblast phenotypes and cardiac fibrotic remodeling in response to insults such as myocardial ischemia.

In the present study, we achieved myofibroblast-specific ablation of LRRC8A via using Cre recombinase driven by the periostin (Postn) promoter in the *Lrrc8a*^flox/flox^ mice (CF-KO). Utilizing these mouse models and *in vitro* cell experiments, we demonstrate for the first time that LRRC8A plays a critical role in the regulation of fibroblast into myofibroblast transformation and myocardial fibrotic remodeling following MI. From the translational perspective, these results reveal the therapeutic role of LRRC8A inhibition in attenuating myocardial fibrosis and HF.

## Methods

Please see the Supplemental Materials (**File S1**) for the detailed methods and materials. The antibody and primer information is available in **Table S1** and **Table S2**.

### Myofibroblast-Specific Ablation of LRRC8A

All the experiments involving the use of animals were approved by the Animal Use and Care Committee of the Fourth Military Medical University, Xi'an, China. The *Lrrc8a*^flox/flox^ mice were established by the clustered regularly interspaced short palindromic repeats (CRISPR)/Cas9 (Cyagen Biosciences Inc., Guangzhou, China). The exon 3 of the *Lrrc8a* gene was selected as the conditional KO region and the gRNA target sequences are as follows. gRNA1 (*Lrrc8a* reverse strand match): TGAGATAGTCCATGTAGCCCTGG. gRNA2 (*Lrrc8a* forward strand match): CAAAGTCGGGCTTAGATGGGTGG. To establish the conditional myofibroblast-specific LRRC8A knockout (CF-KO) mice, the *Lrrc8a*^flox/flox^ mice were crossed with tamoxifen-inducible periostin-Cre (PostnMCM) mice (No. 029645, Jackson Laboratory, Marine, US). At 8-12 wk of age, the *Lrrc8a*^flox/flox^/PostnMCM mice were given a tamoxifen hydrochloride-contained chow diet (400 mg/kg, Harlan Teklad, US) for 28 d, followed by a regular chow diet for another 15 d to ensure the clearance of tamoxifen from the animals 18. *Lrrc8a*^flox/flox^/PostnMCM/tamoxifen mice were the CF-KO, while *Lrrc8a*^flox/flox^/tamoxifen littermates were the wild-type (WT) control.

### Statistical Analysis

All the data are presented as the mean ± SD. GraphPad Prism 8 software (GraphPad Software Inc., San Diego, US) was used to analyze the data. The differences between two groups were compared by a two-tailed unpaired Student's t test. For comparisons of more than two groups, 1-way or 2-way ANOVA followed by a Bonferroni post hoc test was performed as appropriate. A *P* value of less than 0.05 was considered statistically significant.

## Results

### LRRC8A was upregulated in ischemic fibrotic hearts and fibroblasts

**Figure 1:**
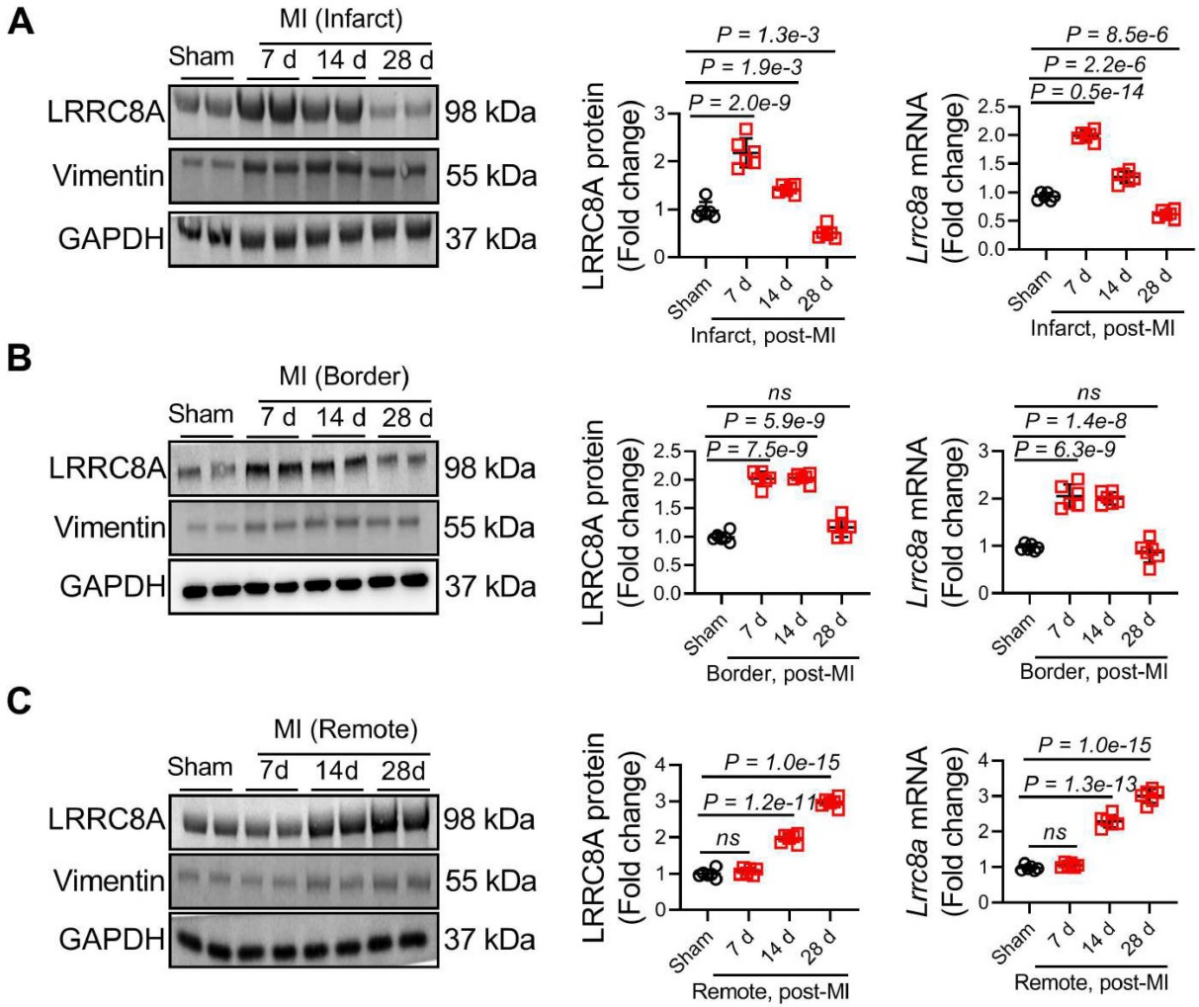
** Upregulation of leucine-rich repeat-containing protein 8A (LRRC8A) in the ischemic heart and cardiac fibroblast**. Wild-type (WT) mice were subjected to sham or myocardial infarction (MI) surgery at 8-12 wk of age. (A) Western blotting and real-time quantification PCR were performed on the heart lysates from the infarct zones at indicated time points. (B) Western blotting and real-time quantification PCR were performed on the heart lysates from the border zones at indicated time points. (C) Western blotting and real-time quantification PCR were performed on the heart lysates from the remote zones at indicated time points. The protein expression of vimentin (a marker of fibroblasts) was set up as the positive control of the fibrotic area. n = 5 mice per group. Data were analyzed by 1-way ANOVA followed by Bonferroni post hoc test.

To test the hypothesis of whether LRRC8A regulates myofibroblast phenotypes and myocardial fibrosis, we first determined the effect of MI on cardiac LRRC8A expression. The expression of vimentin (a marker of fibroblasts) was simultaneously measured as the positive control of the fibrotic area. Adult WT mice were subjected to an MI operation, and left ventricular (LV) lysates from the infarct, border, and remote zone were analyzed for LRRC8A expression at different time points as indicated. In the infarct zone, the expression of LRRC8A peaked on 7 d and gradually decreased on 14 and 28 d post-MI (**Figure 1A**). In the border zone, MI induced maximal LRRC8A expression on 7 d following MI and slowly declined at later time points (**Figure 1B**). In the remote zone, the upregulation of LRRC8A expression was observed on 14 d and continued to increase during the observation period (**Figure 1C**). These observations suggest that the upregulation of LRRC8A is associated with pronounced fibrogenesis. To further confirm the localization of the observed MI-related LRRC8A upregulation, cells were isolated from Sham and MI hearts on 28 d after the operation, and the expression of LRRC8A was determined immediately upon cell isolation. The results showed that the LRRC8A protein expression was markedly increased in the fibroblast isolated from MI hearts, whereas the expression of LRRC8A in cardiac myocytes remained unchanged (**Figure S1A**). Moreover, the co-immunofluorescent staining showed a significant increase in the number of LRRC8A and vimentin double-positive cells in the infarct, border, and remote zone on 14 d post-MI, a time-point at which the elevation of LRRC8A was observed by Western blot in all the injured or fibrotic areas (**Figure S1B**). These results indicate that LRRC8A expression was significantly induced in cardiac fibroblasts by MI.

### Myofibroblast-specific LRRC8A KO mouse models were established

To specifically evaluate the role of myofibroblast LRRC8A in MI-related fibrotic remodeling and HF, we established a mouse model in which LRRC8A was conditionally ablated in the myofibroblasts. Postn is an ECM protein that is abundantly produced in activated myofibroblasts rather than other cell types in sites of cardiac damage, according to genetic lineage tracing research 19-21. PostnMCM mice have thus been routinely employed to manipulate gene expression in activated myofibroblasts in the injured heart 22-23. We crossed tamoxifen-inducible PostnMCM mice with *Lrrc8a* ^flox/flox^ mice, who were fed a tamoxifen-contained diet, to get conditional myofibroblast-specific LRRC8A KO mice (CF-KO, **Figure S2A**). The *Lrrc8a*^flox/flox^ littermates who received tamoxifen treatment were set up as the WT controls (**Figure S2A**). Notably, tamoxifen treatment resulted in a 70% reduction of LRRC8A protein in cardiac fibroblasts isolated from CF-KO mice when compared with the WT controls following MI. In contrast, LRRC8A protein levels in the cardiac myocytes were unchanged between the CF-KO and WT group (**Figure S2B**). Consistently, tamoxifen treatment induced a specific reduction of LRRC8A expression in cardiac fibroblasts rather than in cardiac myocytes in CF-KO mice following MI (**Figure S2C**). These results indicate the successful establishment of mouse models with myofibroblast-specific LRRC8A ablation. During the experimental period, CF-KO mice were viable, fertile, reproduced at expected Mendelian ratios, and showed no observable pathological phenotypes.

### LRRC8A ablation in myofibroblasts improved chamber dilatation and ventricular dysfunction after MI

**Figure 2:**
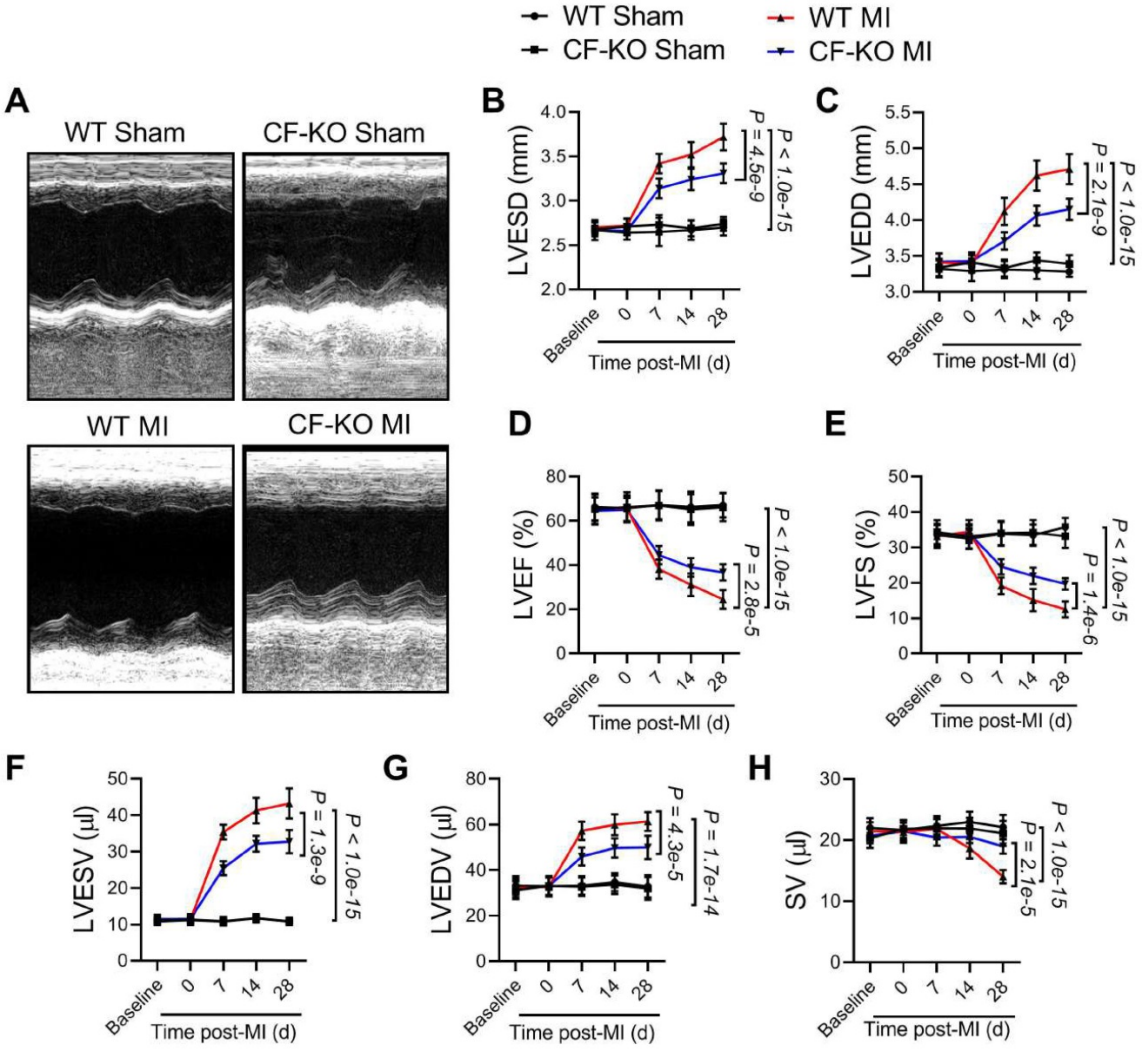
** Ablation of leucine-rich repeat-containing protein 8A (LRRC8A) in myofibroblasts ameliorates ventricular enlargement and dysfunction after myocardial infarction (MI).** Eight to twelve wk-old wild-type (WT) and *Lrrc8a*^flox/flox^ mice bred with PostnMCM mice to generate myofibroblast-specific LRRC8A knockout (CF-KO) mice underwent baseline transthoracic echocardiographic examination. Twenty-four hours later, these mice were subjected to Sham or MI operation. Mice were then followed with serial echocardiography at the time points indicated. (A) Representative M-mode images from 4 wk after MI are shown. (B) Left ventricular end-systolic diameters (LVESD). (C) Left ventricular end-diastolic diameters (LVEDD). (D) Left ventricular ejection fractions (LVEF). (E) Left ventricular fractional shortenings (LVFS). (F) Left ventricular end-systolic volume (LVESV). (G) Left ventricular end-diastolic volume (LVEDV). (H) Stroke volume (SV). n = 7 mice per group. Data were analyzed by 1-way ANOVA followed by Bonferroni post hoc test.

To determine the impact of myofibroblast-specific LRRC8A ablation on the infarction size, 10-12 wk-old WT and CF-KO mice were subjected to the MI operation. The infarct sizes were measured 24 h after MI with the use of triphenyl tetrazolium chloride (TTC) staining. The results showed that the infarct size was comparable between the WT and the CF-KO group, suggesting myofibroblast-specific ablation of LRRC8A exerts little impact on acute myocardial injury in response to MI (**Figure S3A**). These results identified that the myocardial infarct sizes between the WT and CF-KO group were equal in the acute phase and excluded the consistency of the coronary artery ligation location. To determine the role of LRRC8A in the regulation of chronic myocardial remodeling, age-matched adult WT and CF-KO mice were subjected to permanent MI. Before the operation, WT and CF-KO hearts had similar chamber diameters and ventricular contractile function as determined by echocardiography (**Figure 2A** to **2E**). Remarkably, as early as 2 wk post-MI, CF-KO animals had a lower LV end-diastolic and end-systolic diameters (LVEDD and LVESD) when compared with the WT group, demonstrating an improvement in dilatative remodeling in the CF-KO mice (**Figure 2B** and **2C**). This phenomenon was accompanied by an observed improvement in LV dysfunction as evidenced by increased LV ejection fractions (LVEF) and fractional shortenings (LVFS) (**Figure 2D** and **2E**). An improvement in left ventricular end-systolic volume (LVESV), left ventricular end-diastolic volume (LVEDV), and stroke volume (SV) was also observed in CF-KO mice after MI when compared with the WT control (**Figure 2F** to **2H**). These results indicate that myofibroblast-specific knockout of LRRC8A ameliorates post-MI ventricular enlargement and dysfunction.

### LRRC8A deletion in myofibroblasts reduced fibrotic remodeling and myofibroblast transformation after MI

**Figure 3:**
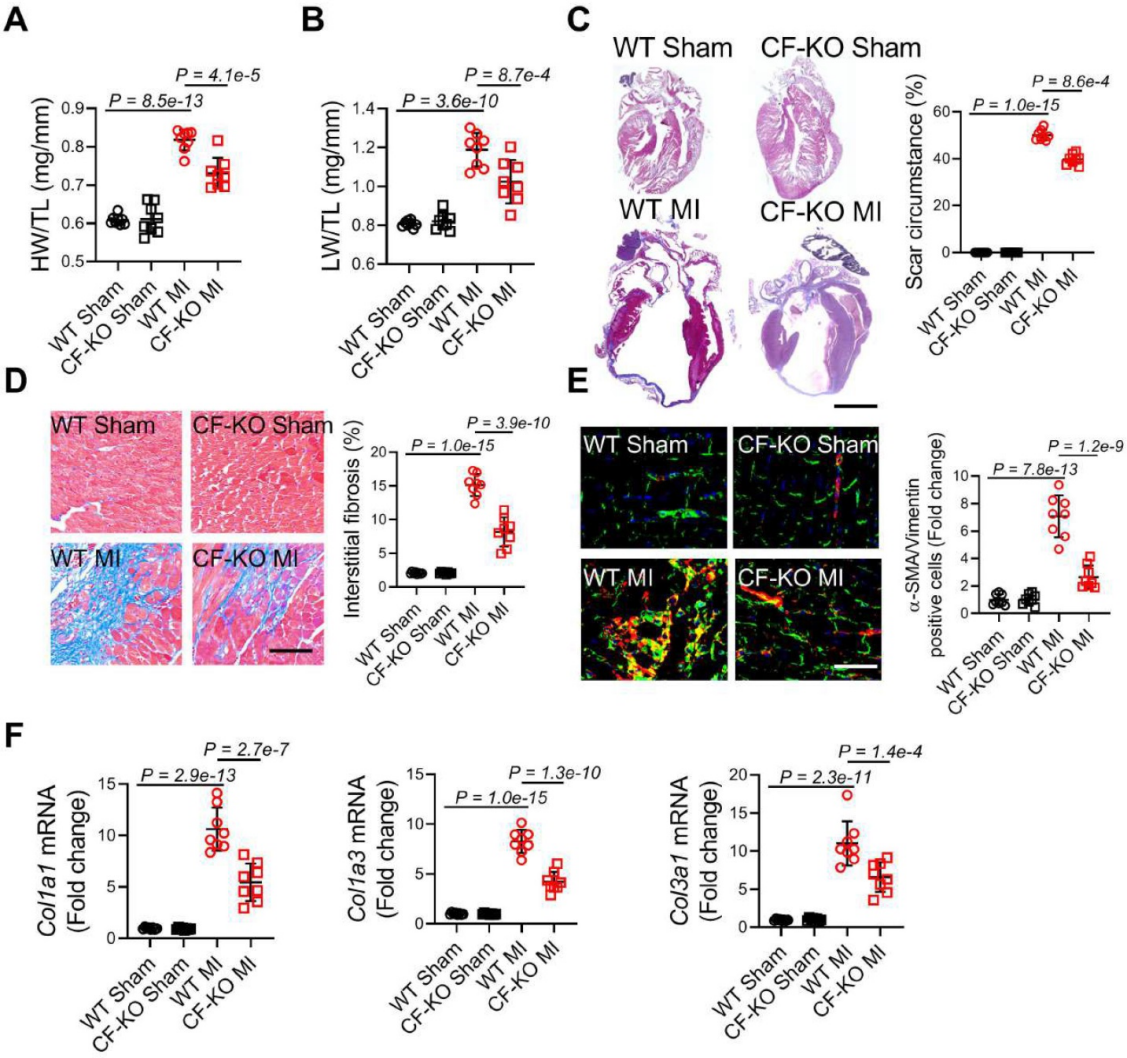
** Ablation of leucine-rich repeat-containing protein 8A (LRRC8A) in myofibroblasts ameliorates scar expansion and interstitial fibrosis.** Eight to twelve wk-old wild-type (WT) and *Lrrc8a*^flox/flox^ mice bred with PostnMCM mice to generate conditional Postn-positive myofibroblast LRRC8A knockout (CF-KO)mice were randomized to receive sham or myocardial infarction (MI) operation for 4 wk. (A) Heart weight to tibia length ratios (HW/TL). (B) Lung weight to tibia length ratios (LW/TL). (C) Representative images of heart sections in long-axis direction stained with Masson trichrome at 4 wk after MI versus sham surgery. Scar circumference was measured and expressed as a percentage of total circumstance of left ventricle. (D) Representative images and quantification of interstitial fibrotic area in left ventricular border zone. (E) Immunofluorescence staining was performed to visualize α-smooth muscle actin (α-SMA, red)/vimentin (green) double-positive cells in the border zone of the post-infarct heart. DAPI (blue) was used to label the nuclei. (F) mRNA expression levels of collagens (*Col1a1*, *Col1a3*, and *Col3a1*) were detected by real-time quantification PCR. n = 7 mice per group. Data were analyzed by 1-way ANOVA followed by Bonferroni post hoc test.

On 28 d after MI, both heart weight to tibial length ratios (HW/TL) and lung weight to tibial length ratios (LW/TL) were significantly decreased in CF-KO mice, demonstrating reduced post-MI cardiac hypertrophy and pulmonary congestion due to HF in CF-KO mice (**Figure 3A and 3B**). Next, we determined the role of cardiac fibroblast LRRC8A in myocardial fibrotic remodeling. Hearts were excised from WT and CF-KO mice on 28 d post-MI and masson trichrome staining was performed. Scar circumference was reduced in CF-KO hearts as compared to the WT group in both long-axis and short-axis direction slices (**Figure 3C** and **Figure S3B**). The interstitial fibrotic area was also significantly reduced in the border zone of CF-KO hearts after MI (**Figure 3D**). Transformation of fibroblasts into myofibroblasts, characterized by expression of α-SMA and deposition of ECM components, is a determinant in fibrotic remodeling. Consistent with the decreased fibrosis and fibrotic scar expansion, we observed a decrease in the number of α-SMA/vimentin double-positive myofibroblasts (**Figure 3E**) and the mRNA expression of collagens (*Col1a1*, *Col1a3*, and *Col3a1*) in the border region of CF-KO hearts (**Figure 3F**). Furthermore, when CF-KO mice were compared to WT controls, pathological cardiac hypertrophy post-MI was significantly reduced, as demonstrated by lower cardiomyocyte cross-sectional area (CSA) and hypertrophic marker gene (*Nppa*, *Nppb*, and *Myh7*) mRNA levels (**Figure S4A** and **S4B**). The improvement in pathological ventricular hypertrophy is a manifestation of the lessened cardiac remodeling induced by myofibroblast-specific LRRC8A deletion.

### Silencing of LRRC8A expression blocked TGF-β1-induced myofibroblast transformation

**Figure 4:**
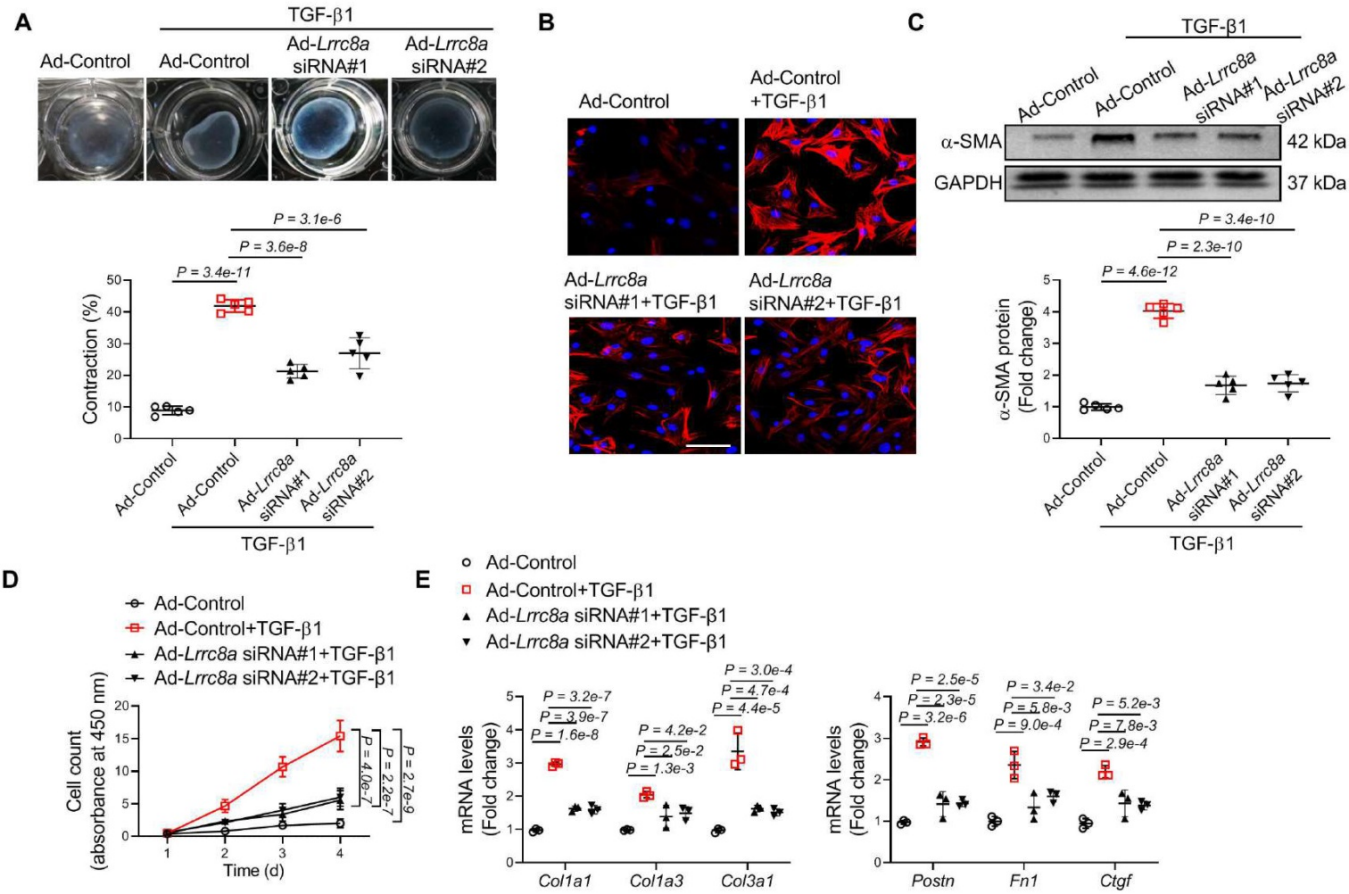
** Adenovirus-mediated leucine-rich repeat-containing protein 8A (LRRC8A) silencing suppresses myofibroblast phenotypes upon transforming growth factor-β1 (TGF-β1) stimulation**. Primary cardiac fibroblasts were isolated from the hearts of neonatal Sprague-Dawley rats. Fibroblasts were transfected with adenovirus vectors carrying small interfering RNA targeting *Lrrc8a* (Ad-*Lrrc8a* siRNA) or empty control (Ad-Control) followed by exposure to vehicle or TGF-β1 (10 ng/mL). (A) Collagen contractility assay with representative collagen gels showing contraction 48 h after the gel release, with percent collagen gel contraction quantified over a 48 h period. (B) Immunofluorescence staining was performed to detect the intensity ofα-smooth muscle actin (α-SMA). (C) Western blotting was performed to detect α-SMA protein expression. (D) The proliferation rate of cardiac fibroblasts was determined by cell count kit-8 assay kit at indicated time points. (E) mRNA levels of collagens (*Col1a1*, *Col1a3*, and *Col3a1*) and myofibroblast marker genes (*Postn*, *Fn1*, and *Ctgf*) were measured by real-time quantification PCR. n = 3 to 5 individual experiments. Data were analyzed by 1-way ANOVA followed by Bonferroni post hoc test.

TGF-β1 is a cytokine that essentially promotes the transformation of quiescent fibroblasts into activated myofibroblasts. We observed that TGF-β1 treatment significantly upregulated LRRC8A protein and mRNA levels in cardiac fibroblasts, consistent with the *in vivo* observation that LRRC8A expression was increased in cardiac fibroblasts isolated from the fibrotic area (**Figure S5A** and** S5B**). We asked whether LRRC8A participated in TGF-β1-induced myofibroblast activation. To test the hypothesis, primary mouse cardiac fibroblasts were transfected with adenovirus vectors carrying siRNA specific for the Lrrc8a gene (Ad-*Lrrc8a* siRNA) or an empty control (Ad-Control). Ad-*Lrrc8a* siRNA significantly decreased the protein expression of LRRC8A in cardiac fibroblasts when compared to cells transfected with adenovirus carrying an empty vector (Ad-Control, **Figure S6**). These cells were cultured in collagen gels and stimulated with TGF-β1 to induce activation. Time-dependent contraction of the collagen gels was observed in cardiac fibroblasts treated with TGF-β1. This phenomenon was markedly attenuated in cells transfected with Ad-*Lrrc8a* siRNA vectors (**Figure 4A**). Furthermore, the induction of the contractile protein a-SMA confers contractile properties to activated myofibroblasts. We found that TGF-β1 induction of a-SMA expression was significantly reduced in cells transfected with Ad-*Lrrc8a* siRNA, as evidenced by both immunofluorescence and Western blot (**Figure 4B** and **4C**). High proliferative capability is a key characteristic of myofibroblasts. In response to TGF-β1, LRRC8A knockdown significantly slowed down the proliferation of fibroblasts (**Figure 4D**). The mRNA expression levels of collagens (*Col1a1*, *Col1a3*, and *Col3a1*) and myofibroblast markers (*Postn*, *Fn1*, and *Ctgf*) were also attenuated in cardiac fibroblasts transfected with *Lrrc8a* siRNA upon TGF-β1 exposure (**Figure 4E**). Taken together, these data suggest that LRRC8A participates in myofibroblast transformation and that its inhibition is sufficient to suppress TGF-β1-induced cardiac fibroblast activation.

### Overexpression of LRRC8A promoted TGF-β1-induced myofibroblast transformation via LRRD-dependent manner

**Figure 5:**
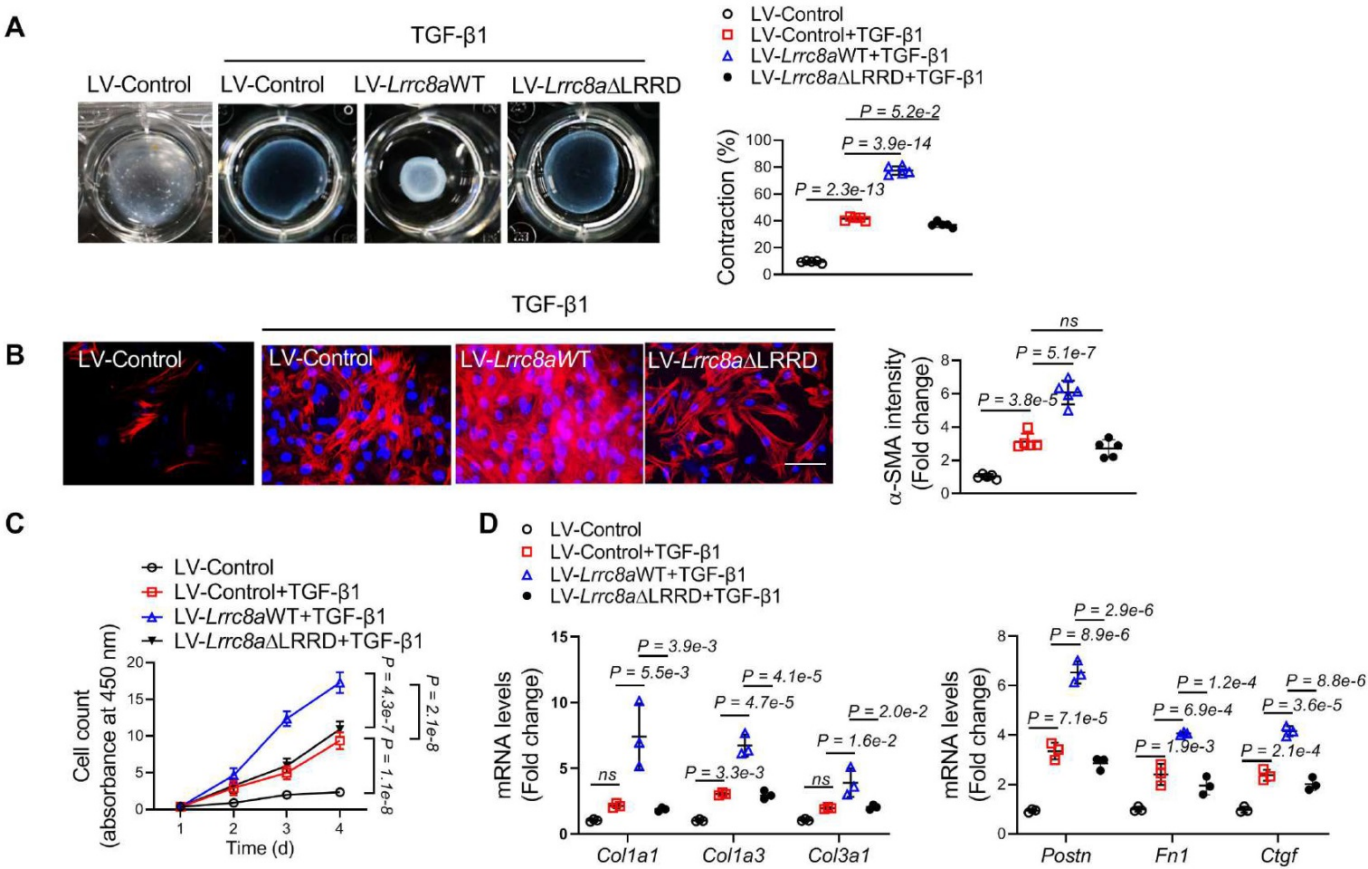
** Leucine-rich repeat-containing protein 8A (LRRC8A) facilitates transforming growth factor-β1 (TGF-β1)-induced myofibroblast transformation via its C-terminal leucine-rich repeat-domain (LRRD).** Cardiac fibroblasts were isolated from the hearts of neonatal Sprague-Dawley rats and were transfected with lentivirus vectors carrying rat wild-type full length *Lrrc8a* gene (LV-*Lrrc8a*WT), mutated *Lrrc8a* gene without its C-terminal LRRD (LV-*Lrrc8a*ΔLRRD), or empty control (LV-Control), respectively. These cells were exposed to vehicle or TGF-β1 (10 ng/mL) to induce myofibroblast transformation. (A) Collagen contractility assay with representative collagen gels showing contraction 48 h after the gel release, with percent collagen gel contraction quantified over a 48 h period. (B) Immunofluorescence staining was performed to detect the intensity of α-smooth muscle actin (α-SMA). (C) The proliferation rate of cardiac fibroblasts was determined by cell count kit-8 assay kit at indicated time points. (D) mRNA levels of collagens (*Col1a1*, *Col1a3*, and *Col3a1*) and myofibroblast marker genes (*Postn*, *Fn1*, and *Ctgf*) were measured by real-time quantification PCR. n = 3 to 5 individual experiments. Data were analyzed by 1-way ANOVA followed by Bonferroni post hoc test.

To further validate the role of LRRC8A in myofibroblast transformation, cardiac fibroblasts were transfected with lentivirus vectors carrying WT *Lrrc8a* (LV-*Lrrc8a*WT) or empty control (LV-Control) followed by exposure of TGF-β1. Overexpression of LRRC8A facilitated TGF-β1-induced contractile phenotypes in cardiac fibroblasts as evidenced by collagen gel contraction assay (**Figure 5A**). The expression of a-SMA induced by TGF-β1 was increased in LRRC8A overexpressing fibroblasts as determined by both immunofluorescence (**Figure 5B**). LRRC8A overexpression significantly accelerated the proliferation of fibroblasts in response to TGF-β1 (**Figure 5C**). The mRNA expression levels of collagens (*Col1a1*, *Col1a3*, and *Col3a1*) and myofibroblast marker genes (*Postn*, *Fn1*, and *Ctgf*) were also increased in cardiac fibroblasts overexpressed LRRC8A upon TGF-β1 exposure (**Figure 5D**). These results suggest that LRRC8A overexpression facilitates the acquisition of myofibroblast phenotypes upon TGF-β1 exposure.

LRRC8A regulates multiple cellular processes and mediates signaling transduction via its C-terminal LRRD, which does not rely on its anion channel function 14, 17. We next asked whether LRRD is involved in LRRC8A's regulation of myofibroblast transformation. Cardiac fibroblasts were transfected with lentivirus vectors carrying mutated LRRC8A whose C-terminal LRRD was deleted (LV-*Lrrc8a*ΔLRRD). In contrast to the LV-*Lrrc8a*WT group,* Lrrc8a*ΔLRRD overexpression had little impact on TGF-β1-induced myofibroblast phenotypes, as determined by collagen gel contraction assay, a-SMA induction, proliferation assay, as well as collagen and myofibroblast marker gene expression (**Figure 5A** to **5D**). Collectively, these results provide the first evidence that the C-terminal LRRD is required for the facilitating effects of LRRC8A on myofibroblast transformation.

### LRRC8A promoted the acquisition of myofibroblast phenotypes by activating JAK2-STAT3 signaling pathway

**Figure 6:**
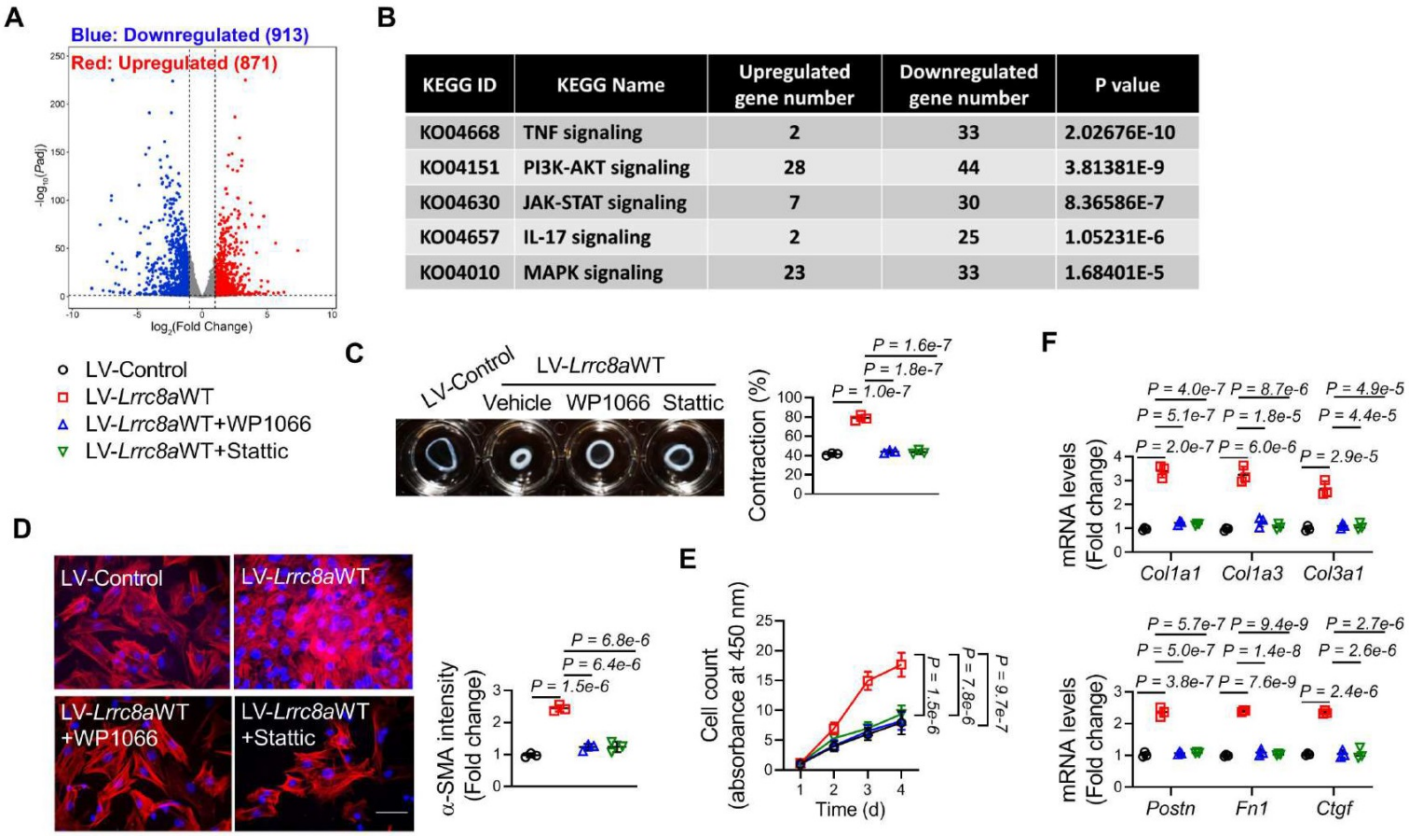
** Leucine-rich repeat-containing protein 8A (LRRC8A) facilitates transforming growth factor-β1 (TGF-β1)-induced myofibroblast transformation via activating the Janus kinase-2 (JAK2)/ signal transducer and activator of transcription-3 (STAT3) pathway.** (A) Primary cardiac fibroblasts were isolated from the hearts of neonatal Sprague-Dawley rats. Fibroblasts were transfected with adenovirus vectors carrying small interfering RNA targeting *Lrrc8a* (Ad-*Lrrc8a* siRNA) or empty control (Ad-Control) followed by exposure to TGF-β1 (10 ng/mL) for 48 h. Total RNA samples were collected and sequenced. Differentially expressed genes (DEGs) between two groups were shown in the Volcano Plot (Log2|fold change|>=1 and *P*_adjust_ <0.05). (B) The most significantly changed Kyoto Encyclopedia of Genes and Genomes (KEGG) pathways were shown. (C) Fibroblasts were transfected with lentivirus vectors carrying rat wild-type full length *Lrrc8a* gene (LV-*Lrrc8a*WT) or empty control (LV-Control) followed by exposure to TGF-β1 (10 ng/mL), co-treated with vehicle, WP1066 (a specific JAK2 inhibitor, 10 nmol/L) or Stattic (a specific STAT3 inhibitor, 10 nmol/L). Collagen contractility assay with representative collagen gels showing contraction 48 h after the gel release, with percent collagen gel contraction quantified over a 48 h period. (D) Immunofluorescence staining was performed to detect the intensity of α-smooth muscle actin (α-SMA). (E) The proliferation rate of cardiac fibroblasts was determined by cell count kit-8 assay kit at indicated time points. (F) mRNA levels of collagens (*Col1a1, Col1a3,* and *Col3a1*) and myofibroblast marker genes (*Postn, Fn1,* and *Ctgf*) were measured by real-time quantification PCR. n = 3 individual experiments. Data were analyzed by 1-way ANOVA followed by Bonferroni post hoc test.

We next explored the downstream mechanism underlying LRRC8A regulation of myofibroblast transformation. RNA sequencing (RNA-seq) was performed using the RNA samples collected from primary cardiac fibroblasts transfected with Ad-Control or Ad-*Lrrc8a* siRNA in response to TGF-β1 treatment (**File S2**). RNA-seq identified 1784 differentially expressed genes (DEGs, Log2|fold change|>=1 and *P*_adjust_ <0.05) between these two groups (**Figure 6A**). Among these DEGs, 913 genes were upregulated, whereas 871 genes were downregulated in cardiac fibroblasts transfected with Ad-Lrrc8a siRNA (**Figure 6B**). Kyoto Encyclopedia of Genes and Genomes (KEGG) analysis of the DEGs revealed that the Janus kinase (JAK)-Signal transducer and activator of transcription (STAT) signaling pathway (KO04630) ranked as one of the most significantly changed pathways in fibroblasts transfected with Ad-*Lrrc8a* siRNA (**Figure 6C**). Among the top-ranked signaling pathways, persistent JAK2-STAT3 pathway activation is a predominant driver of myofibroblast transformation and maintenance^20^-^21^. Thus, we focused on this pathway. LRRC8A silencing resulted in the downregulation of 30 genes in JAK-STAT signaling pathway (**Table S3**). Among them, the downregulation of STAT3 downstream genes, such as *Socs3*, *Il6*, and *Pim1*, was further confirmed by RT-PCR in fibroblasts transfected with Ad-*Lrrc8a* siRNA, supporting that the JAK2-STAT3 signaling pathway is inactivated in LRRC8A silenced cardiac fibroblasts (**Figure S7A**). In TGF-β1-treated fibroblasts, the phosphorylation (activation) levels of JAK2 (T1007/1008) and STAT3 (T705) were significantly reduced by LRRC8A silencing when compared to the control group (**Figure S7B**). In contrast, the phosphorylation (activation) levels of JAK2 and STAT3 were further elevated in the fibroblasts overexpressing LRRC8A (**Figure S7C**). These results suggest that LRRC8A regulates the activation of the JAK2-STAT3 signaling pathway during myofibroblast transformation induced by TGF-β1.

To further identify the involvement of the JAK2-STAT3 signaling pathway in LRRC8A regulation of myofibroblast phenotypes, the JAK2-STAT3 pathway was inhibited in fibroblasts overexpressing LRRC8A by WP1066 (the selective inhibitor of JAK2) or Stattic (the selective inhibitor of STAT3), respectively. The facilitating effects of LRRC8A overexpression on TGF-β1-induced myofibroblast phenotypes were totally abolished by the inhibition of the JAK2-STAT3 pathway, as evidenced by collagen gel contraction assay, α-SMA induction, proliferation assay, as well as collagen and myofibroblast marker gene expression (**Figure 6C** to** 6F**). Collectively, these results provide direct evidence demonstrating that LRRC8A facilitates TGF-β1-induced myofibroblast transformation via activating the JAK2-STAT3 signaling pathway.

### LRRD/GRB2/JAK2 Interaction was required for LRRC8A-induced JAK2-STAT3 pathway activation

**Figure 7:**
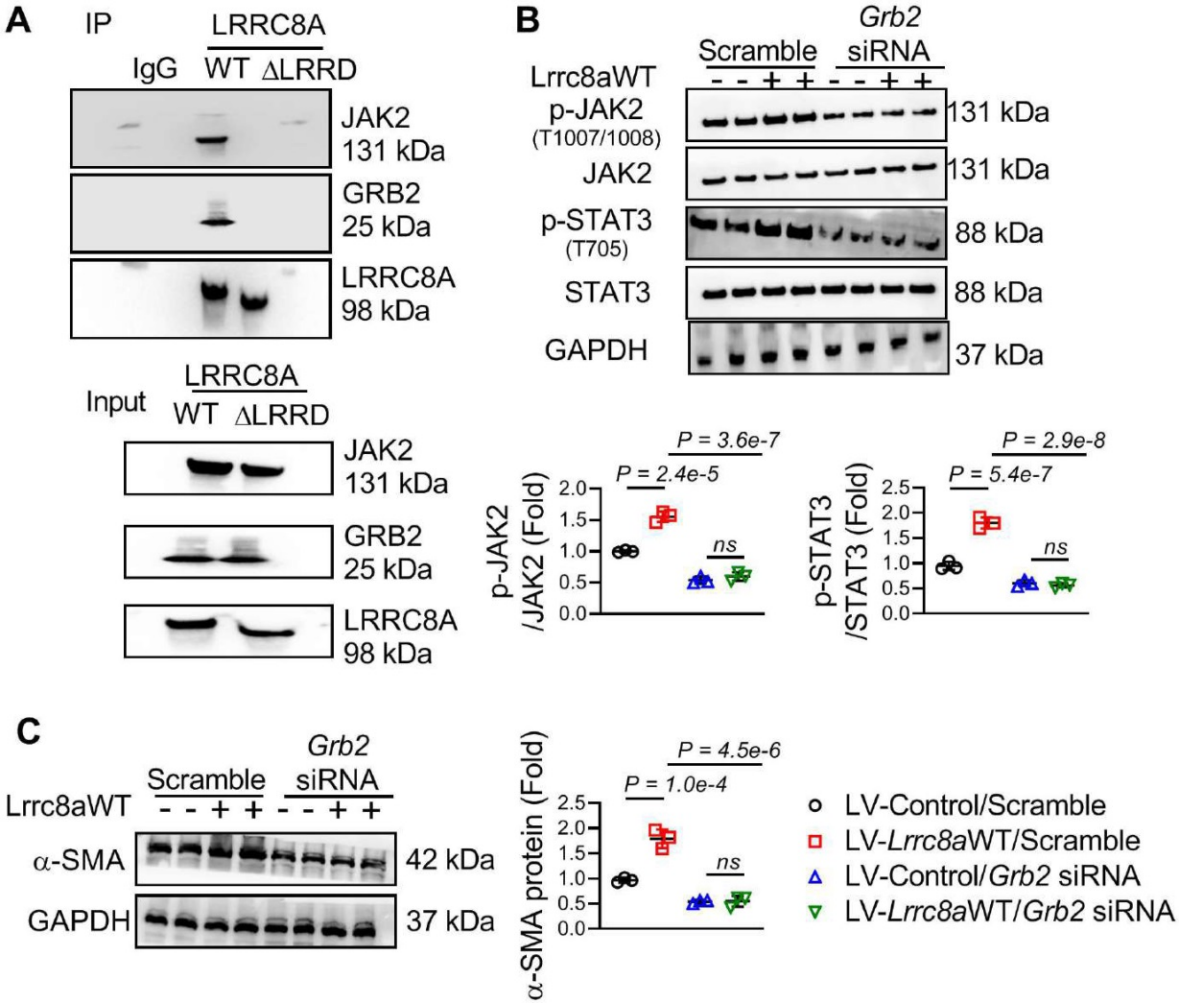
** Leucine-rich repeat-containing protein 8A (LRRC8A) interacts with Janus kinase-2 (JAK2) and growth factor receptor-bound protein 2 (GRB2) to regulate signal transducer and activator of transcription-3 (STAT3) activation and myofibroblast phenotypes.** (A) Fibroblasts were transfected with lentivirus vectors carrying rat wild-type full length *Lrrc8a* gene (LV-*Lrrc8a*WT), mutated Lrrc8a gene without its C-terminal leucine-rich repeat-domain (LV-*Lrrc8a*ΔLRRD), or empty control (LV-Control), respectively. Co-immunoprecipitation assay showed that LRRC8A interacts with JAK2 and GRB2 in dependent of its C-terminal LRRD. (B) Fibroblasts were transfected with LV-*Lrrc8a*WT or LV-Control, co-transfected with scramble or* Grb2* siRNA. These cells were next exposed to TGF-β1 (10 ng/mL) for 48 h. Western blotting was performed to detect p-JAK2, JAK2, p-STAT3, STAT3, and GAPDH protein levels. (C) Western blotting was performed to detect α-smooth muscle actin (α-SMA) and GAPDH protein levels. n = 3 individual experiments. Data were analyzed by 1-way ANOVA followed by Bonferroni post hoc test.

Growth factor receptor-bound protein 2 (GRB2) combines with the C-terminal LRRD of LRRC8A to regulate intracellular signaling transduction 14-15. The co-immunoprecipitation assay further showed that LRRC8A interacted with JAK2 and GRB2, whereas this interaction was absent when the LRRD was deleted from LRRC8A (**Figure 7A**). This observation suggests that LRRC8A interacts with GRB2 and JAK2 via its C-terminal LRRD. Overexpression of LRRC8A facilitated the activation of the JAK2-STAT3 signaling pathway in TGF-β1-stimulated fibroblasts, whereas this effect disappeared when the LRRD of LRRC8A was deleted (**Figure S7C** and** S7D**). These results supported the notion that LRRD is essential for LRRC8A's regulation of the JAK2-STAT3 signaling pathway. To establish the role of GRB2 in LRRC8A-induced JAK2-STAT3 activation, GRB2 expression was silenced by siRNA (**Figure S8**). Intriguingly, the facilitating effect of LRRC8A overexpression on the activation of the JAK2-STAT3 signaling pathway was greatly weakened by GRB2 silencing in LV-*Lrrc8a*WT transfected fibroblasts upon TGF-β1 treatment (**Figure 7B**). As a consequence, GRB2 silencing prevented the facilitation of LRRC8A overexpression on TGF-β1-induced myofibroblast transformation, as evaluated by the expression of a-SMA (**Figure 7C**). Collectively, these results reveal for the first time that LRRC8A-LRRD/GRB2/JAK2 constitutes a protein complex facilitating the activation of the JAK2-STAT3 signaling pathway and participates in TGF-β1-stimulated myofibroblast transformation.

## Discussion

In the present study, we have made a series of novel findings. First, we have identified that LRRC8A is a key modulator of myofibroblast transformation and cardiac fibrosis. Cardiac fibroblasts are the predominant cell type regulating the myocardial fibrotic process upon pathological stimuli, such as pressure overload and ischemic insults 2. The transformation of quiescent fibroblasts into activated myofibroblasts is generally recognized as the key cellular step resulting in interstitial fibrosis, ventricular compliance loss, and HF progression 3. In mammalian cells, LRRC8A is a protein component of VRAC 6-7. A substantial body of evidence suggests that LRRC8A regulates a wide range of cellular processes, including cell growth, proliferation, and survival, independent of its VRAC function 8-10. However, the role of LRRC8A in cardiovascular physiopathology remains elusive. Here, we found that the expression of LRRC8A was much higher in the cardiac fibroblasts than in the cardiac myocytes. Thus, we established the myofibroblast-specific LRRC8A knockout mice and revealed that the deletion of LRRC8A in the fibroblasts limited the post-MI fibrotic remodeling and HF progression. *In vitro* experiments showed that LRRC8A was required for the transformation of fibroblasts into myofibroblasts upon TGF-β1. These* in vivo* and* in vitro* data for the first time emphasize that the restriction of LRRC8A expression is a promising therapeutic strategy for fibrotic remodeling and HF post-MI.

Second, we have demonstrated that the JAK2-STAT3 signaling pathway underlies the LRRC8A-mediated regulation of myofibroblast transformation. Upon revealing the key role of LRRC8A in regulating myofibroblast phenotypes, RNA-Seq was performed to screen the potential signaling pathway underlying the biological effects of LRRC8A. KEGG analysis showed that the JAK-STAT pathway ranked as one of the most significantly changed pathways in fibroblasts transfected with Ad-Lrrc8a siRNA. Persistent activation of the JAK2-STAT3 signaling pathway results in the upregulated expression of fibrotic gene programs and promotes the transformation of fibroblasts into myofibroblasts 24-25. In the present study, we observed that overexpression of LRRC8A enhanced, whereas silencing of LRRC8A weakened, the activation of the JAK2-STAT3 signaling pathway in TGF-β1 stimulated myofibroblasts. Moreover, pharmacological inhibition of JAK2 or STAT3 significantly blocked the facilitating effect of LRRC8A overexpression on TGF-β1-induced myofibroblast transformation. These results demonstrate that the JAK2-STAT3 signaling pathway is responsible for the LRRC8A modulation of myofibroblast transformation.

Third, we have found that the C-terminal LRRD of LRRC8A is required for the modulation of the canonical JAK2-STAT3 pathway in myofibroblasts. LRRC8A has been identified as a molecule regulating signaling pathway transduction in various types of cells independent of its VRAC function 26. LRRC8A-regulated signaling pathway transduction is mainly through its C-terminal LRRD, which interacts with a variety of intracellular adaptor proteins, including GRB2 27. GRB2 is an adaptor protein associated with and necessary for tyrosine-phosphorylated JAK2 28. In the present study, we have demonstrated that LRRC8A overexpression facilitated, whereas silencing of LRRC8A weakened, the activation of the JAK2-STAT3 signaling pathway and the myofibroblast phenotypes upon TGF-β1 stimulation 29-30. Intriguingly, this biological effect of LRRC8A totally disappeared when its C-terminal LRRD was deleted. In the molecular aspects, the interaction of LRRC8A, JAK2, and GRB2 was totally lost when LRRC8A lost its LRRD. These results suggest that LRRC8A, JAK2, and GRB2 constitute a protein complex that facilitates the activation of the JAK2-STAT3 pathway and promotes the transformation of cardiac fibroblasts into myofibroblasts, providing novel insights into the role of LRRC8A in regulating intracellular signaling pathway transduction.

## Conclusions

In summary, we have identified a novel and central role for LRRC8A in the regulation of myocardial fibrotic remodeling after MI. LRRC8A exerts these effects in cardiac fibroblasts via a brand-new molecular mechanism involving its C-terminal LRRD, the adaptor protein GRB2, and the JAK2-STAT3 signaling pathway. From the translational perspective, the present study reveals the promising therapeutic potential of LRRC8A inhibition in suppressing cardiac fibrosis in response to detrimental stresses such as ischemic insults.

## Supplementary Material

Supplementary methods, figures and tables.

Supplementary file 2.

## Abbreviations

α-SMA: α-smooth muscle actin
ECM: extracellular matrix
GRB2: growth factor receptor-bound protein 2
HF: heart failure
HW/TL: heart weight to tibial length ratios
JAK: Janus kinase
KEGG: Kyoto Encyclopedia of Genes and Genomes
KO: knockout
LRRC8A: leucine-rich repeat-containing protein-8A
LRRD: leucine-rich repeat-domain
LV: left ventricular
LW/TL: lung weight to tibial length ratios
MI: myocardial infarction
Postn: periostin
STAT: signal transducer and activator of transcription
TGF-β1: transforming growth factor-β1
VRAC: volume regulatory anion channel
WT: wild-type
