# Supplementary material for: LRRC8A critically regulates myofibroblast phenotypes and fibrotic remodeling following myocardial infarction
Source: Theranostics. 2022 Jul 27;12(13):5824–35. doi: 10.7150/thno.75200 (PMC9373806; doi:10.7150/thno.75200)
Supplement: Supplementary file 1 — Supplementary methods, figures and tables. [file thnov12p5824s1.pdf]

LRRC8A critically regulates myofibroblast phenotypes and fibrotic remodeling following myocardial infarction

Running title: ***LRRC8A Modulates Post-MI Fibrosis***

Xiyao Chen[1]*, Fuyang Zhang[2]*, Guangyu Hu[2]*, Xing Li[1], Lin Wang[1],Congye Li[2], Cong Huo[1], Rong Xu[1], Liming Hou[1], Ning Wang[1], Xiaoming Wang[1]#.

1. Department of Geriatrics, Xijing Hospital, Fourth Military Medical University, Xi'an 710032, China.
2. Department of Cardiology, Xijing Hospital, Fourth Military Medical University, Xi'an 710032, China.

*These authors contributed equally.

1. Supplemental materials and methods
2. Supplemental tables: 3
3. Supplemental figures: 8

#Corresponding author:

Xiaoming Wang, MD.
Department of Geriatrics, Xijing Hospital, Fourth Military Medical University,
No. 127 Changle West Road, Xi'an 710032, China

E-mail: xmwang@fmmu.edu.cn

Supplemental materials and methods

Animals and myocardial infarction models

The animal experiment procedures in the present study were approved by the Animal Care and Use Committee of the Fourth Military Medical University and adhered to the National Institutes of Health Guidelines for the Use of Laboratory Animals. *Lrrc8a*$^{flox/flox}$ mice were established by the clustered regularly interspaced short palindromic repeats (CRISPR)/Cas9 (Cyagen Biosciences, Guangzhou, China) and crossed with tamoxifen-inducible periostin-Cre (PostnMCM) mice (Jackson Laboratory, Maine, US). At 8-12 wk of age, mice were given a tamoxifen -contained chow diet (400 mg/kg, Harlan Teklad, US) for 28 d followed by a regular chow diet for another 15 d to ensure the clearance of tamoxifen from the animals [1]. The *Lrrc8a*$^{flox/flox}$/PostnMCM/tamoxifen mice were conditionally knockout (CF-KO), whereas the *Lrrc8a*$^{flox/flox}$/tamoxifen littermates represented wild-type (WT) controls.

To establish MI models, age-matched (8 to 12-wk-old) male WT and CF-KO mice were anesthetized by inhalation of isoflurane (1.5%, YiPin Pharmaceutical, Shijiazhuang, China) and left anterior descending coronary artery ligation operation was performed as we previously described [2]. To establish the MI models, the WT and CF-KO mice (n = 20 per group) were anesthetized by inhaling 1.5% isoflurane. A purse suture was placed over a tiny skin cut (approximately 1 cm) on the left chest. The 4th intercostal gap was disclosed when the pectoral major and minor muscles were dissected and retracted. To open the pleural membrane and pericardium, a small hole was cut at the 4th intercostal gap with a mosquito clamp. The heart was "popped out" smoothly and softly with the clamp slightly open. Using a 6-0 silk suture, the left coronary artery was located, sutured, and ligated at a point ~3 mm from its origin. When the anterior wall of the LV turned pale, the ligation was pronounced successful. Following ligation, the heart was immediately returned to the intra-thoracic space, followed by manual evacuation of air and closure of muscle and skin using the previously inserted purse-string suture.

In the peri-operation period and the following observational period of 28 d post-MI, the mortality rates were 45% (9/20) in the WT group and 25% (5/20) in the CF-KO group, respectively. At the indicated time points after MI, the animals were scarificed and the hearts were harvested. The border zone of the MI-operated heart was defined as the immediate neighboring regions around the infarction as seen under the stereomicroscope. The remote zone contained areas ~2-3 mm away from the infarct region and the posterior wall of the left ventricle. Before the tissue collection and analysis, the border and remote zone were stereologically identified.

Echocardiography

Mice were anesthetized by the inhalation of 1.5% isoflurane. M-mode echocardiography was performed by a VisualSonics 770 echo system (FUJIFILM VisualSonics, Toronto, Canada) as we previously described [2]. Left ventricles (LV) were fully viewed in the short-axis between the two papillary muscles. Measurements were performed by the results of 3 continuous heartbeats. LV end-systolic diameters

(LVESD), LV end-diastolic diameters (LVEDD), LV end-systolic volume (LVESV), and LV end-distolic volume (LVEDV) were measured. Left ventricular ejection fractions (LVEF) and LV fractional shortenings (LVFS) were calculated as follows: LVEF=[(LVEDV-LVESV)/LVEDV] ×100% and LVFS=[(LVEDD-LVESD)/LVEDD] ×100%.

Isolation and culture of adult mouse cardiac fibroblasts

Adult cardiac fibroblasts were isolated from 8-12-wk-old WT mice. Hearts were rapidly excised and washed by ice-cold phosphatase saline buffer (PBS). The tissues were rinsed in ice-cold Hank's balanced salt solution and digested in type II collagenase (100 U/ml, Sigma Aldrich, Shanghai, China) at 37 ℃ for 20 min. The first digestion was discarded and the medium collected from the second digestion containing the cardiac fibroblasts was centrifuged for 5 min at 3000 rpm and resuspended in DMEM (Thermo Fisher, Shanghai, China) supplied with 10 % fetal bovine serum (FBS, Thermo Fisher, Shanghai, China). This procedure was repeated until the digestion medium was transparent. Cells were plated in 60 mm dishes and attached for 1 h before the first media change to remove non-adherent cells, including myocytes and endothelial cells.

Isolation of adult mouse cardiac myocytes

Adult mouse cardiac myocytes were isolated as we previously described [3]. In brief, the mice were anesthetized with 1.5% isoflurane and fully heparinized with heparin. Hearts were carefully removed and placed into ice-cold PBS. Next, the heart was attached to the Langendorff system via the aorta and was fully perfused with perfusion solution (126 mmol/L NaCl, 4.4 mmol/L KCl, 18 mmol/L $NaHCO_3$, 1 mmol/L $MgCl_2$, 11 mmol/L glucose, 10 mmol/L 2,3-butanedione monoxime, 30 mmol/L taurine, and 4 mmol/L HEPES) for 5 min. The heart was then perfused by the collagenase solution (perfusion solution with 0.1% bovine serum albumin, 0.025 mmol/l $CaCl_2$ and 0.1% type II collagenase) for another 10 min. After full digestion, the ventricle was triturated with a 10 ml pipette at a slow speed and filtered through a 100 µm filter.

Isolation and culture of neonatal rat cardiac fibroblasts

Neonatal cardiac fibroblasts were isolated from 1- to 3-d-old Sprague-Dawley rats. Briefly, the rat's heart was quickly removed and 0.04% collagenase II (Sigma Aldrich, Shanghai, China) was used to digest the minced ventricles 4 to 6 times. Cells were harvested and suspended in DMEM medium enriched with 10% FBS. Cardiac fibroblasts were isolated and collected by using the differential time adherent method. These cells were cultured in DMEM supplied with 10% FBS. Passage 2 cells were used for the following experiments.

Adenovirus transfection

For adenoviral infection of cardiac fibroblasts, we employed replication-defective adenovirus vectors. The adenoviruses were constructed by

Hanbio Biotech, Shanghai, China. Adenovirus expressing GFP was used to control for nonspecific effects of adenoviral infection (Ad-Control). Two independent adenovirus vectors carrying 2 distinct siRNAs targeting *Lrrc8a* were transfected into fibroblasts to knock down LRRC8A protein expression. The siRNA sequences were as follows: siRNA#1 5'-GCTGATGATTGCTGTCTTTGG-3'; siRNA#2 5'-GCCTTCATGTTGCACCTCATC-3'. At 24 h after plating, cardiac fibroblasts were infected with the adenovirus vectors diluted in DMEM medium (MOI=100). The medium was replaced with virus-free SFM medium 24 h following adenoviral infection, and cells were cultured for an additional 12 h prior to experiments.

Lentivirus transfection

Lentiviruses carrying rat wild-type full-length *Lrrc8a* gene (LV-*Lrrc8a*WT), mutated rat *Lrrc8a* gene with deletion of its C-terminal LRRD (LRRC8A del 91/+26aa, LV-*Lrrc8a*ΔLRRD), and empty control (LV-Control) were constructed by Genechem Co.,Ltd., Shanghai, China. At 24 h after plating, cardiac fibroblasts were infected with lentiviruses diluted in DMEM medium (MOI=200). Polybrene (5 μg/ml) was added to enhance the transfection efficacy. The medium was replaced with virus-free SFM medium 24 h following adenoviral infection, and cells were cultured for an additional 48 h prior to experiments.

Western blotting

Tissue or cell lysates were collected in the RIPA buffer (P0013B, Beyotime, Beijing, China) in the presence of 1% protease/phosphatase inhibitor cocktails (#5872, Cell Signaling Tech, Shanghai, China). Proteins were separated on SDS-PAGE gels and transferred to polyvinylidene difluoride (PVDF) membranes (Merck Millipore, Darmstadt, Germany). Membranes were incubated overnight at 4 ℃ with primary antibodies. After washing by phosphate saline buffer with 0.1% tween 20, blots were incubated at room temperature for 1 h with horseradish peroxidase (HRP)-conjugated goat anti-mouse or goat anti-rabbit secondary antibodies (1:5,000, Cowin Biotechnology, Beijing, China). Proteins were visualized using an enhanced chemiluminescence kit (Merck Millipore, Darmstadt, Germany). The blot was scanned by the ChemiDocXRS equipment (Bio-Rad Labratories, Shanghai, China), and densities of the bands were analyzed by Quantity One software (Bio-Rad Labratories, Shanghai, China). The detailed information about primary antibodies is listed in **Table S1.**

Real-time quantification PCR

Total RNA was extracted from frozen tissues or cultured cells by the MiniBEST Universal RNA Extraction Kit (#9767, Takara, Dalian, China). RNA was reversely transcribed into cDNA using the PrimeScript™ RT reagent kit with gDNA Eraser (DRR047A, Takara, Dalian, China). RT-PCR was performed in triplicate using a PCR detection kit (DRR081A, Takara, Dalian, China) and a CFX96 system (Bio-Rad Labratories, Shanghai, China). The primer sequences used in the present study are available in **Table S2**.

RNA sequencing

Cardiac fibroblasts were transfected with Ad-Control or Ad-*Lrrc8a* siRNA and treated with TGF-β1 (10 ng/ml) for 48 h. RNA library preparation, sequencing, and analysis were performed by Personalbio Tech Ltd. (Shanghai, China). A total of 3 μg of RNA per sample was utilized for RNA library preparation with the NEBNext® UltraTM RNA Library Prep Kit for Illumina® (NEB, USA). The library was sequenced on an Illumina NovaSeq6000 platform. After quality control, reads were aligned to the reference genome using the STAR read mapper. Then, HTSeq v0.6.0 software was used to count the read numbers mapped to each gene. Differentially expressed genes (DEGs) were identified using the DESeq2 package (Log2|fold change|≥1 and P adj<0.05). Kyoto Encyclopedia of Genes and Genomes (KEGG) analysis in DEGs was performed. The detailed information about DEGs in the JAK-STAT Signaling Pathway is shown in **Table S3**. The whole RNA sequencing data is available in **File S2**.

Immunofluorescence staining

Cells were fixed with 4% paraformaldehyde for 10 min and permeablized with 0.4% Triton 100 (Beyotime, Beijing, China) in phosphate saline buffer (PBS) for 10 min at room temperature. After being rinsed with PBS, cells were blocked with 2% bovine serum albumin (BSA) in PBS for 1 h at room temperature and incubated overnight with primary antibodies at 4 ℃. Cells were then stained with secondary antibodies for 1 h following 4',6-diamidino-2-phenylindole (DAPI) staining for another 3 min at room temperature. Images were acquired with a confocal microscope (LSM880, Carl Zeiss, Germany) using the same exposure parameters. Negative controls stained with only secondary antibodies were included in all experiments. Images were analyzed using ImageJ. Fixed tissues were sectioned at a thickness of 5 μm following a series of deparaffinization and dehydration. Slides were then subjected to antigen retrieval in a hot citric acid buffer. After cooling, slides were permeabilized, blocked, incubated with primary antibodies, incubated with secondary antibodies, and stained DAPI as described before. Images were scanned by Pannoramic MIDI (3D HISTECH) and analyzed using ImageJ. The detailed information of the primary antibodies are listed in **Table S1**.

Co-immunoprecipitation

Cardiac fibroblasts were transfected with LV-Control, LV-*Lrrc8a*WT, and LV-*Lrrc8a*ΔLRRD. Cells were harvested in a cell lysis buffer. After protein quantification, 500 μg of protein was incubated with 4 μg of antibodies against LRRC8A extracellular epitope (Alomone, Israel) overnight at 4 ℃ using a rotator followed by another 2-h incubation with Protein G Plus/Protein A agarose beads (Cell Signaling Technology, Shanghai, China). Beads were washed 3 times with ice-cold PBS, protein complexes were released by 5 min of boiling in SDS sample buffer, resolved by SDS-PAGE, and subjected to immunoblotting.

Small interfering RNA (siRNA) transfection

siRNA targeting rat *Grb2* and scramble control were designed, synthesized, and validated by Genechem Co.,Ltd. (Shanghai, China). *Grb2* siRNA sequence was the follows. GRB2 siRNA: 5'-GTACAAGGCAGAGCTTAATTT-3'. The siRNA transfection was performed using Lipofectamine RNAi^{MAX} according to the manufacturer's instructions (Invitrogen) with minor modification. Briefly, 5×10^6 cells were transfected in 2 ml of SFM containing 500 μl Opti-MEM (Invitrogen, Shanghai, China), 8 μl Lipofectamine RNAi^{MAX}, and 100 nmol/L of siRNA.

Collagen gel contraction assay

The ability of fibroblasts to contract collagen was assessed using the Cell Contraction Assay (#CBA-201, Cell Biolabs, San Diego, US) per the manufacturer's protocol. Fibroblasts were combined with a collagen solution, which was then allowed to polymerize. The collagen gel was detached from the plate and collagen gel size change (contraction index) was measured at various times and quantified with the ImageJ software.

Masson trichrome staining

Myocardium tissues were fixed with 4% paraformaldehyde for 24 h, dehydrated by increasing concentrations of ethanol, and embedded in paraffin. Tissue sections (5 μm) were stained with Masson trichrome staining kit (Sigma Aldrich, Shanghai, China) as the manufacturer's instruction described.

Wheat germ agglutinin (WGA) staining

Myocardium tissues were fixed with 4% paraformaldehyde for 24 h, dehydrated by increasing concentrations of ethanol, and embedded in paraffin. Tissue sections (5 μm) were stained with WGA staining kit (ServiceBio, Wuhan, China) as the manufacturer's instruction described.

2,3,5-triphenyltetrazolium chloride (TTC) staining and the infarction size measurement

The heart was rapidly removed from the mouse and washed by ice-old PBS. The heart was put on the solidified carbon dioxide for 15 min to freeze. The heart was cut into 1 mm thick slices along the atrioventricular sulcus from the apex to the bottom. The heart was cut into five pieces. The pieces were quickly placed into 5 ml 1.5% TTC buffer (pH = 7.4) for 15 min at 37 °C. TTC staining showed that the infarcted area was white and the non-infarcted area was red. The white/red area ratio at the papillary muscle surface was calculated by ImageJ software and recognized as the infarction size.

References

1. Lal H, Ahmad F, Zhou J, Yu JE, Vagnozzi RJ, Guo Y, et al. Cardiac fibroblast glycogen synthase kinase-3beta regulates ventricular remodeling and dysfunction in ischemic heart. Circulation. 2014;130(5): 419-30.

2. Zhang F, Xia Y, Yan W, Zhang H, Zhou F, Zhao S, et al. Sphingosine 1-phosphate signaling contributes to cardiac inflammation, dysfunction, and remodeling following myocardial infarction. Am J Physiol Heart Circ Physiol. 2016; 310(2): H250-61.

3. Li Y, Xiong Z, Yan W, Gao E, Cheng H, Wu G, et al. Branched chain amino acids exacerbate myocardial ischemia/reperfusion vulnerability via enhancing GCN2/ATF6/PPAR-α pathway-dependent fatty acid oxidation. Theranostics. 2020; 10(12):5623-40.

Supplemental tables

Table S1. Primary antibody information

Target	Supplier	Catalog	Usage	Dilution
LRRC8A	CST	#24979	WB, IF	1:500 (WB), 1:50 (IF)
LRRC8A	Alomone Labs	AAC-001	IP	8 μg/mg protein lysate
α-SMA	CST	#19245	WB, IF	1:1000 (WB), 1:100 (IF)
p-JAK2	Abclonal	AP0531	WB	1:1000
JAK2	Abclonal	A19629	WB	1:1000
p-STAT3	Abclonal	AP0705	WB	1:1000
STAT3	Abclonal	A19566	WB	1:1000
GRB2	Abclonal	A19059	WB	1:1000 (WB)
Vimentin	CST	#5741	WB, IF	1:1000 (WB), 1: 200 (IF)
GAPDH	Abclonal	A19056	WB	1:3000

Table S2. Primer sequence information

Gene name	Forward (5’-3’)	Reverse (5’-3’)
Lrrc8a	ACTCCTGCAACGACTCCTTC	ACTGGTGTCGATCTAGGTCATAC
Col1a1	AGGGCGAGTGCTGTGCTTT	CCCTCGACTCCTACATCTTCTGA
Col1a3	TGAAACCCCAGCAAAACAAAA	TCACTTGCACTGGTTGATAAGATT AA
Col3a1	CCCTGGACCTCAGGGTATCA	GGGTTTCCATCCCTTCCAGG
Postn	TGGTATCAAGGTGCTATCTGCG	AATGCCCAGCGTGCCATAA
Fn1	ACCGAAGCCGGGAAGAGCA A	GGTCCGTTCCCACTGCTGATTTAT C
Ctgf	CTGCGAGGAGTGGGTGTG	ATGTGTCTTCCAGTCGGTAGG
Nppa	GCTTCCAGGCCATATTGGAG	GGGGGCATGACCTCATCTT
Nppb	GAGGTCACTCCTATCCTCTGG	GCCATTTCCTCCGACTTTTCTC
Myh7	ACTGTCAACACTAAGAGGGTCA	TTGGATGATTTGATCTTCCAGGG
Gapdh	ACCACAGTCCATGCCATCAC	TCCACCACCCTGTTGCTGTA

Table S3. Differentially expressed genes in JAK-STAT signaling pathway between Ad-Control and Ad-*Lrrc8a* siRNA group

Gene ID	Ad-Control	Ad-*Lrrc8a* siRNA	Fold change	P-value	Gene name
ENSRNOG00000000145	87.49134118	222.5110921	2.543235583	6.24414E-08	*Pik3r3*
ENSRNOG00000000875	577.6936815	1885.237021	3.263385218	7.50245E-32	*Fhl1*
ENSRNOG00000002919	3.193384588	14.68520432	4.598633177	0.003405065	*Gfap*
ENSRNOG00000005747	29.72161992	66.75308429	2.245943675	0.000108012	*Il27ra*
ENSRNOG00000011973	57.71639369	139.2792385	2.413165993	8.7029E-05	*Il7*
ENSRNOG00000015654	216.8865848	460.7569129	2.124414072	3.43351E-16	*Ghr*
ENSRNOG00000018962	32.92337412	68.94714647	2.094170124	0.000891445	*Ctf1*
ENSRNOG00000000187	3957.263326	1258.12006	0.317926798	7.2251E-25	*Csf2rb*
ENSRNOG0000000	8116.416682	3841.068393	0.473246821	2.84147E-26	*Cdkn1a*

0521					
ENSRNOG00000000529	1536.524992	484.8748693	0.315565885	5.99642E-42	*Pim1*
ENSRNOG00000002244	21988.00063	8305.228416	0.377716399	1.99581E-48	*Pdgfra*
ENSRNOG00000002946	3045.647011	559.0599277	0.183560316	2.22442E-61	*Socs3*
ENSRNOG00000003254	119.1371115	7.981193771	0.066991668	1.00039E-13	*Il23a*
ENSRNOG00000003954	469.0472515	180.1116552	0.383994693	2.32761E-14	*Il2rg*
ENSRNOG00000004332	1207.876953	583.1332589	0.482775383	1.7087E-08	*Egfr*
ENSRNOG00000004380	11.09136841	0	0	1.04763E-05	*Il12b*
ENSRNOG00000004647	101.1896102	5.054098141	0.049946809	1.2434E-15	*Il10*
ENSRNOG00000006268	26.07415502	4.22553034	0.162058189	4.42664E-06	*Ifnb1*
ENSRNO	11574.44942	617.2348215	0.053327	1.81801	*Lif*

G0000000 7002			359	E-41	
ENSRNO G0000000 8525	1802.371939	23.58828411	0.013087 356	1.13335 E-32	*Csf3*
ENSRNO G0000000 9468	20.19603388	5.762863427	0.285346 294	0.002514 319	*Il12a*
ENSRNO G0000001 0278	3796.099222	171.1052725	0.045073 973	2.41099 E-20	*Il6*
ENSRNO G0000001 2619	18.95411663	7.312426777	0.385796 232	0.019596 396	*Epor*
ENSRNO G0000001 5441	5708.615595	2753.578143	0.482354 802	6.73212 E-11	*Il4r*
ENSRNO G0000001 6384	1367.852512	584.9542406	0.427644 235	2.58507 E-35	*Pik3cb*
ENSRNO G0000001 7197	57.12642453	28.29652209	0.495331 579	0.002762 768	*Pdgfb*
ENSRNO G0000001 7386	515.1947199	16.53241114	0.032089 636	1.34293 E-10	*Il11*
ENSRNO G0000001 8076	105.9141616	42.96179204	0.405628 401	0.000184 539	*Fmo5*

ENSRNOG00000019496	1222.655804	490.6409729	0.401291166	1.48365E-17	*Stat5a*
ENSRNOG00000024390	310.0408005	58.55363315	0.188857831	1.00823E-14	*Osm*
ENSRNOG00000025571	16.97702344	0.927311193	0.054621542	0.000758054	*Il19*
ENSRNOG00000026805	542.6096614	9.551301953	0.017602528	3.53806E-49	*Csf2*
ENSRNOG00000029543	563.7920962	133.1564555	0.236180068	6.47685E-53	*Cish*
ENSRNOG00000033192	9922.580262	4456.923336	0.449169794	3.6108E-26	*Osmr*
ENSRNOG00000033984	660.5642148	288.2687792	0.436397814	1.0512E-23	*Ifnlr1*
ENSRNOG00000047647	71.23140172	3.371603003	0.047333099	4.36347E-23	*Il2ra*
ENSRNOG00000059968	21623.96397	7869.865721	0.363941862	5.43669E-57	*Jak2*

Supplemental figures and figure legends

Figure S1

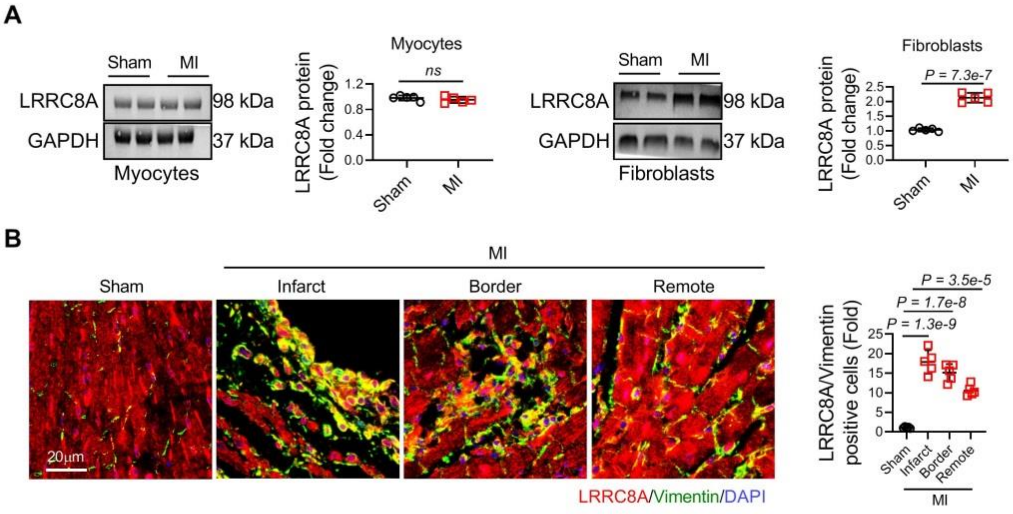

Figure S1. LRRC8A expression was upregulated in cardiac fibroblasts in the infarcted heart. (A) LRRC8A protein expression was measured in cardiac myocytes and fibroblasts isolated from the sham or MI heart on 28 d after the surgery. n= 5 mice per group. Data were analyzed by unpaired student's t test. (B) Representative images and quantification of LRRC8A (red) and vimentin (green) co-immunofluorescent staining in the infarct, border, and remote area of the infarcted heart. n = 5 mice per group. Data were analyzed by 1-way ANOVA followed by Boferroni post hoc test.

Figure S2

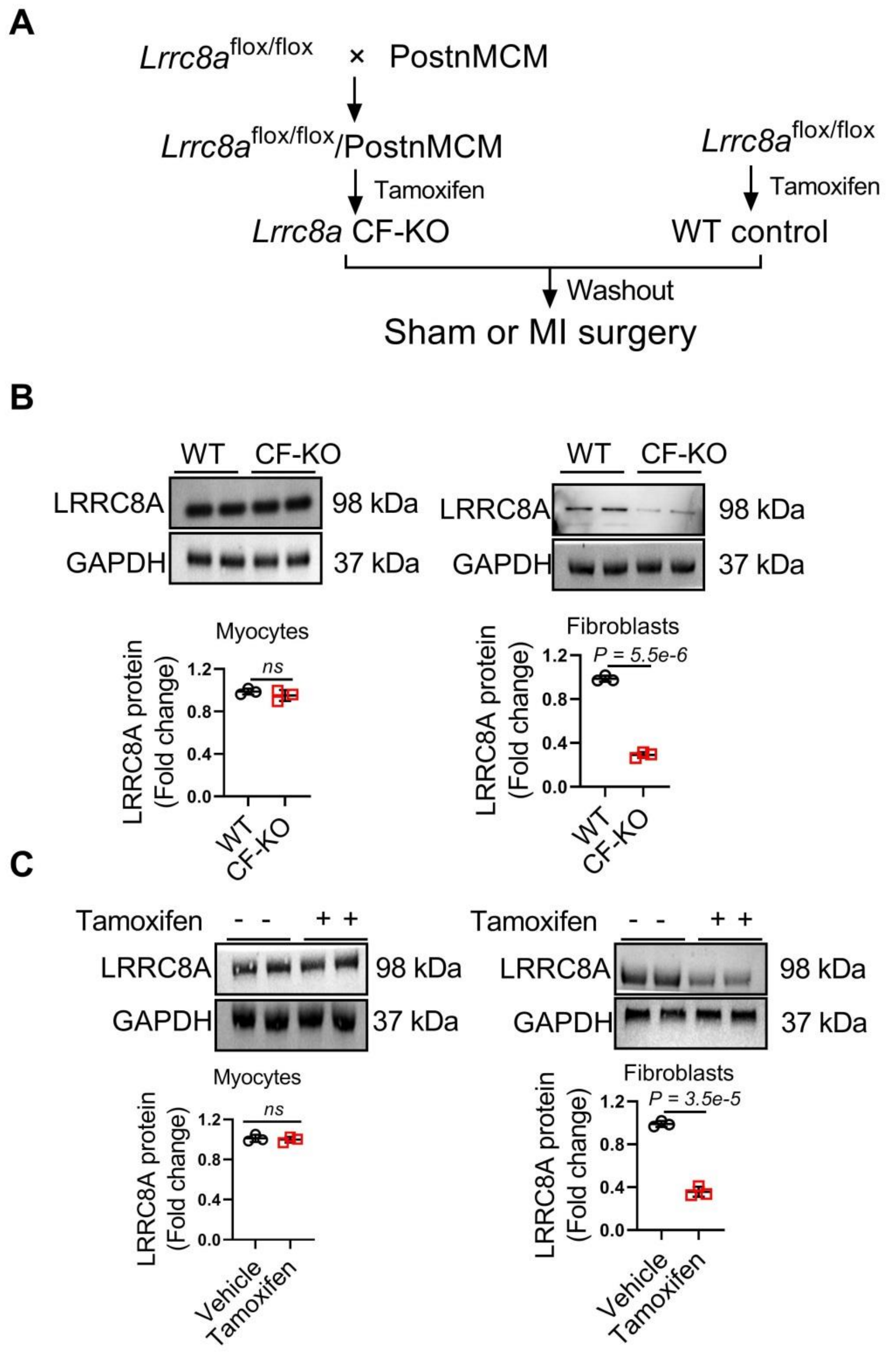

Figure S2. Establishement and validation of the myofibroblast-specific LRRC8A knockout mouse. (A) Illustration of conditional myofibroblast-specific LRRC8A knockout (CF-KO) mouse model establishment. (B) LRRC8A protein was measured in cardiac myocytes and fibroblasts isolated from adult CF-KO or WT mouse hearts after MI. (C) LRRC8A protein was measured in cardiac myocytes and fibroblasts isolated from *Lrrc8a*$^{flox/flox}$/PostnMCM mice with vehicle or tamoxifen treatment. n = 3 mice per group. Data were analyzed by unpaired student's t test.

Figure S3

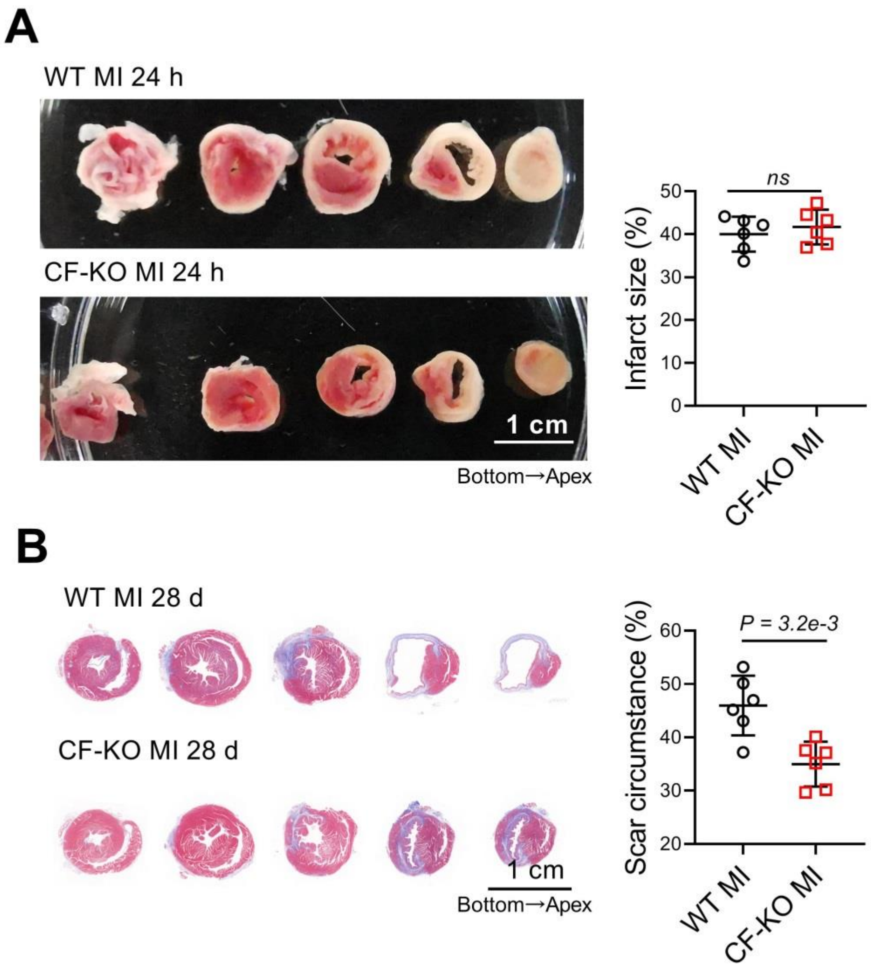

Figure S3. Acute infarct size and chronic fibrotic scar formation in WT and CF-KO mice following MI. (A) WT and CF-KO mice were subjected to MI operation and the infarcted sizes were evaluated by triphenyl tetrazolium chloride (TTC) staining 24 h post-MI. n= 6 mice per group. Data were analyzed by unpaired student's t test. (B) Representative images of heart sections from the bottom to apex in short-axis direction stained with masson trichrome on 28 d after MI. Scar circumference was measured and expressed as a percentage of total circumference of left ventricle. n = 6 mice per group. Data were analyzed by unpaired student's t test.

Figure S4

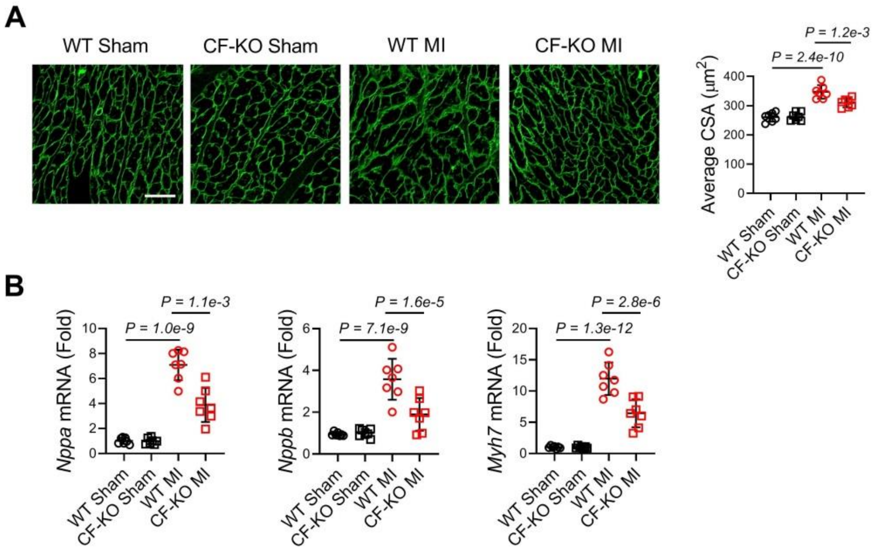

Figure S4. Myocardial hypertrophy in WT and CF-KO mice after MI. (A) Representative images of wheat germ agglutinin (WGA) staining and quantification results of cross-sectional areas (CSA) of cardiac myocytes. The average CSA were calculated by the CSA of all cardiac myocytes in five randomly selected visusal field in the border zone on 28 d post-MI. (B) The mRNA levels of hypertrophic marker gene including *Nppa*, *Nppb*, and *Myh7* were determined by RT-PCR. n = 7 mice per group. Data were analyzed by 1-way ANOVA followed by Bonferroni post hoc test.

Figure S5

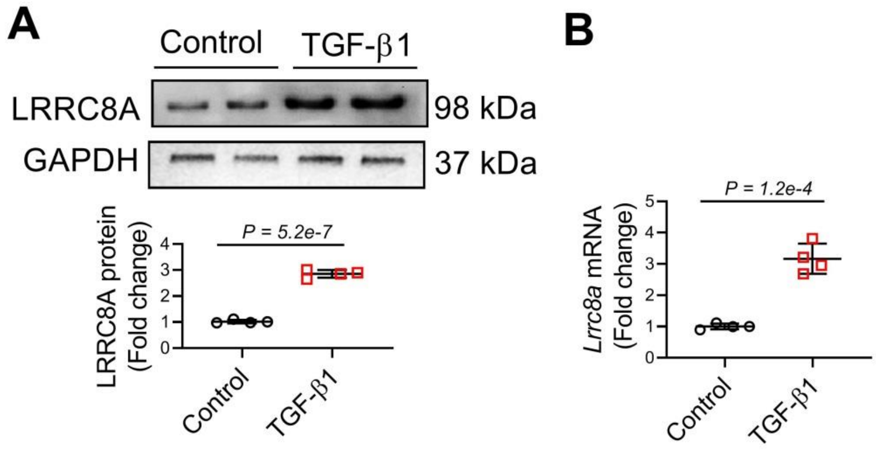

Figure S5. TGF-β1 upregulated LRRC8A expression in cardiac fibroblasts. The protein (A) and mRNA (B) levels of LRRC8A were determined in primary cardiac fibroblasts treated with vehicle or TGF-β1 (10 ng/ml) for 48 h. n = 4 individual experiments. Data were analyzed by unpaired student's t test.

Figure S6

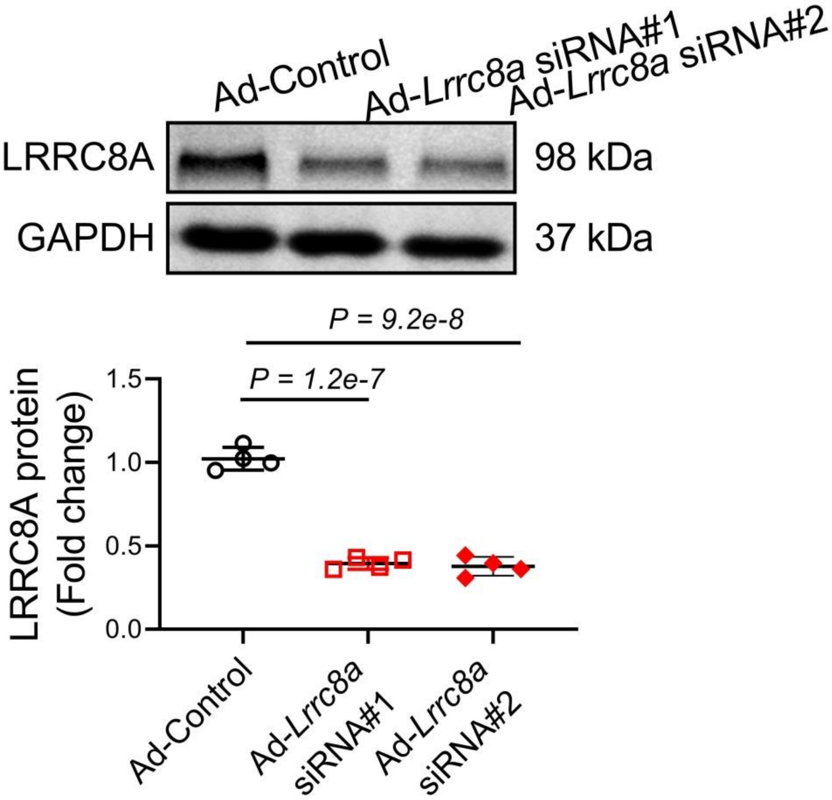

Figure S6. Knockdown efficacy of *Lrrc8a* siRNA in cardiac fibroblasts. Cardiac fibroblasts were transfected with adenovirus vectors carrying two independent *Lrrc8a* siRNAs (Ad-*Lrrc8a* siRNA#1 and #2) or empty control (Ad-Control). 24 h after the transfection, LRRC8A protein was measured by Western blotting. n = 4 individual experiments. Data were analyzed by 1-way ANOVA followed by Bonferroni post hoc test.

Figure S7

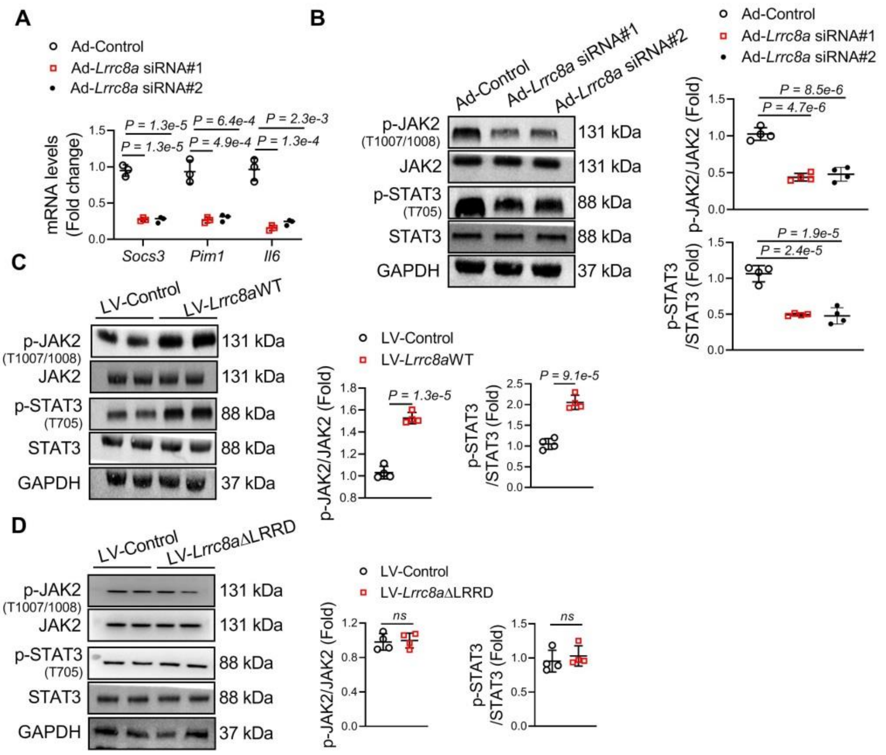

Figure S7. The influence of LRRC8A knockdown or overexpression on the JAK2-STAT3 signaling pathway in cardiac fibroblasts. Cardiac fibroblasts were transfected with adenovirus vectors carrying *Lrrc8a* siRNA (Ad-*Lrrc8a* siRNA#1 or #2) or empty control (Ad-Control). (A) Upon TGF-β1 (10 ng/ml) treatment, mRNA levels of STAT3 target genes including *Socs3*, *Pim1*, and *Il6* were measured by RT-PCR. (B) Upon TGF-β1 (10 ng/ml) treatment, p-JAK2, JAK2, p-STAT3, STAT3, and GAPDH were measured by Western blotting. (C) Cardiac fibroblasts were transfected with lentivirus vectors carrying wild-type rat *Lrrc8a* gene (LV-*Lrrc8a*WT) or empty control (LV-Control). Upon TGF-β1 (10 ng/ml) treatment, p-JAK2, JAK2, p-STAT3, STAT3, and GAPDH were measured by Western blotting. (D) Cardiac fibroblasts were transfected with lentivirus vectors carrying mutated *Lrrc8a* gene without LRRD (LV-*Lrrc8a*ΔLRRD) or empty control (LV-Control). Upon TGF-β1 (10 ng/ml) treatment, p-JAK2, JAK2, p-STAT3, STAT3, and GAPDH were measured by Western blotting. n = 3 to 4 individual experiments. Data were analyzed by unpaired student's t test or 1-way ANOVA followed by Bonferroni post hoc test.

Figure S8

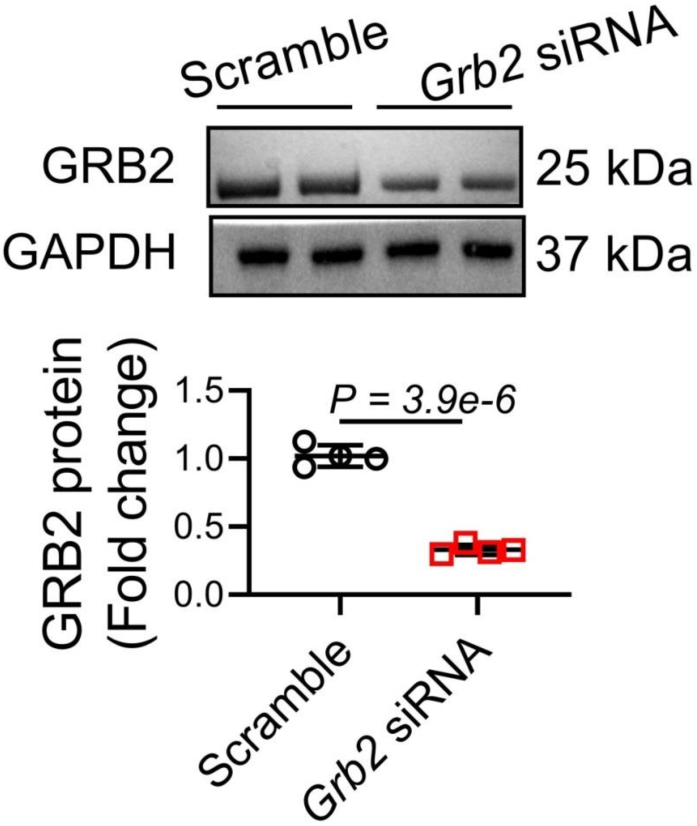

Figure S8. Knockdown efficacy of *Grb2* siRNA in cardiac fibroblasts. Cardiac fibroblasts were transfected with scramble or *Grb2* siRNA. 24 h after the transfection, GRB2 protein was measured by Western blotting. n = 4 individual experiments. Data were analyzed by unpaired student's t test.
